# The relative benefits for environmental sustainability of vegan diets for dogs, cats and people

**DOI:** 10.1371/journal.pone.0291791

**Published:** 2023-10-04

**Authors:** Andrew Knight

**Affiliations:** 1 School of Environment and Science, Nathan Campus, Griffith University, Nathan, QLD, Australia; 2 Faculty of Health and Wellbeing, University of Winchester, Winchester, United Kingdom; University of Illinois, UNITED STATES

## Abstract

Environmental impacts of the livestock sector are proportional to consumption levels. To assess the relative consumption of livestock animals within the diets of dogs, cats and people, this study examined their dietary energy needs within the US in 2020, and globally in 2018. Also studied were US pet food ingredients, and environmental sustainability indicators for plant- and animal-based foods consumed globally. Relative consumptions of average livestock animals were: US: dogs– 17.7%, cats– 2.3%, humans– 80.0%; and globally: dogs– 7.7%, cats– 1.2%, humans– 91.1%. Full transition to nutritionally-sound vegan diets would spare from slaughter the following numbers of terrestrial livestock animals annually (billions): US: dogs– 1.7, cats– 0.2, humans– 7.8, and globally: dogs– 6.0, cats– 0.9, humans– 71.3, as well as billions of aquatic animals in all dietary groups. Very large impact reductions were also associated with land and water use, emissions of greenhouse gases (GHGs), acidifying and eutrophifying gases, and biocide use, in all dietary groups. If implemented globally, nutritionally-sound vegan diets would free up land larger than the following nations: dogs–Saudi Arabia or Mexico, cats–Japan or Germany, humans–Russia–the world’s largest country–combined with India. Such diets would save freshwater volumes greater than all renewable freshwater in the following nations: dogs–Denmark, cats–Jordan, humans–Cuba. Such diets would reduce GHGs by amounts greater than all GHG emissions from following nations: dogs–South Africa or the UK, cats–Israel or New Zealand, humans–India or the entire EU. The numbers of additional people who could be fed using food energy savings associated with vegan diets exceeded the 2018 human populations of the following nations: dogs–the entire European Union, cats–France or the UK, humans–every single nation or collective region on Earth, as defined by the World Bank. All of these estimates are conservative.

## Introduction

Numerous studies (e.g., [1–4]) have demonstrated substantial adverse environmental impacts of the livestock sector globally. These have included the consumption and use of land, water, fossil fuels, fertilizers and pesticides, and the resultant production of greenhouse gases (GHGs), acidifying emissions such as sulfur dioxide (SO_2_), and eutrophifying emissions such as those arising from phosphate (PO_4_^3-^). It is well recognised within such studies of these phenomena, that current and projected future livestock consumption levels are unsustainable, given planetary resource constraints. Accordingly, numerous studies have called for reduction in reliance on livestock produce within human diets (e.g., [4–6]), along with reductions in food waste and overconsumption.

However, the relevant studies have usually assumed that all or most diet-related livestock impacts are attributable to human diets. To date, few studies have considered the relative impacts of dog and cat diets, or attempted to quantify their environmental impacts in comparison to those of human diets. Studies that have done this to a limited degree include [7–11]. Such focus on dogs and cats is warranted: dog and cat diets account for 95% of global pet food sales [12].

Until recently, this was perhaps understandable, due to widespread assumptions that diets other than meat-based were not feasible for dogs and cats, which are considered biologically omnivorous and carnivorous, respectively. It is beyond the scope of this study to examine the nutritional suitability of vegan diets (which exclude any animal products) for dogs and cats. However, recent studies have demonstrated good digestibility of such diets [13, 14]. A considerable body of recent evidence indicates that provided such diets are formulated to be nutritionally-sound, as modern commercial vegan diets usually are [15], dogs and cats maintained on vegan diets can have longevity and health at least equivalent, and in some respects superior, to those maintained on conventional meat-based diets. Such results are evident within studies of health outcomes in both dogs (nine studies: [16–24]) and cats (four studies: [23, 25–27]). Dietary palatability also appears equivalent overall [28]. It has long been established that people can be healthily maintained on nutritionally-sound vegan diets (e.g., [29]). Hence, it is now realistically feasible to examine the potential benefits for environmental sustainability, of nutritionally-sound vegan diets for dogs and cats.

Recent studies have indicated that environmental impacts of dog and cat diets are significant [7–11, 30–32]. This is unsurprising, considering that domestic dogs have a total global biomass of around 20 million tonnes–approximately equal to the combined biomass of all remaining wild terrestrial mammals. Cats have a total biomass of around two million tonnes–almost double that of the African savanna elephant [33]. It has been reported that pets consume about 20% of the world’s meat and fish, and that an area double the size of the UK is used to produce dry pet food for cats and dogs each year [34]. Approximately three million tonnes of fish are consumed within UK pet food annually [34]. In the US, meat produce consumption by dogs and cats appears responsible for up to 80 million tons of methane (CH_4_) and nitrous oxide (N_2_O) production [7].

Despite such results, to date the relevant literature has focused almost exclusively on recommending dietary change for humans. However, we now understand that nutritionally-sound vegan diets are feasible for dogs and cats, and do not compromise pet welfare [28]. Accordingly, it is now important to examine the relative impacts on environmental sustainability of conventional meat-based diets for dogs, cats and people, and to compare the environmental benefits that could be expected to result from nutritionally-sound vegan diets. Hence this study was designed to quantify the relative consumption of livestock and aquatic animals by dogs, cats and people, and the number of such animals who would be spared annually from slaughter, if each group was transitioned on to nutritionally-sound vegan diets. It also aimed to calculate resultant savings in land and water use, and of GHGs, acidifying and eutrophifying emissions, and in biocide use. Finally, it sought to calculate the number of additional people, dogs and cats who could be fed using food energy savings, in light of substantial dietary energy losses within meat-based diets during conversion from plant- to animal-based food ingredients [35].

## Methodology

**Fig 1:**
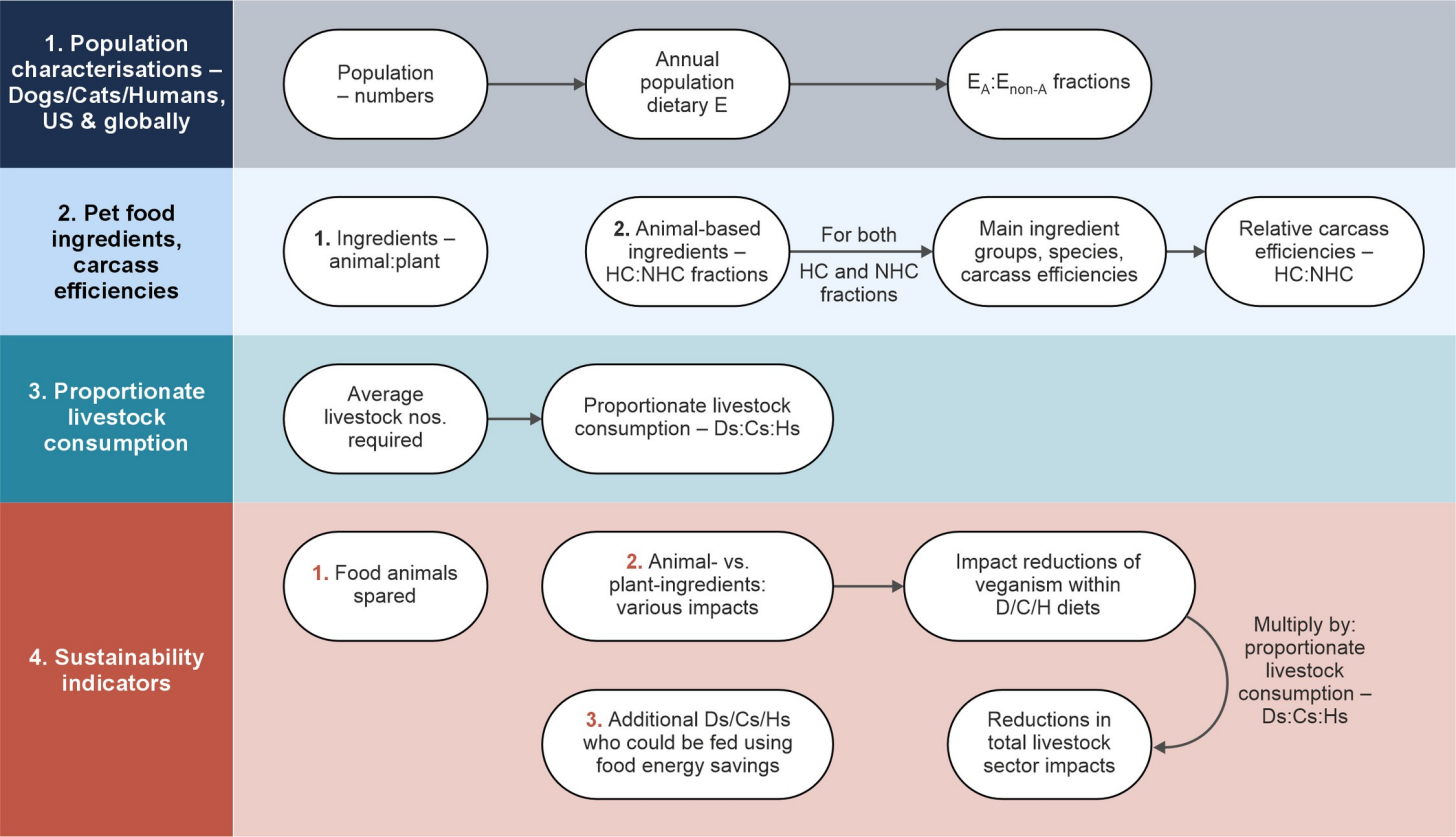
**Study methodological stages.** Note: D = Dog, C = Cat, H = Human, E = dietary energy, E_A_ and E_non-A_ are E from animal and non-animal sources, HC = human-consumable, NHC = non human-consumable.

This study involved four main methodological stages (Fig 1).

### 1. Dog and cat populations within the US and globally

The US was chosen as the initial focus, as it has the largest national population of pet dogs and cats globally, and also, the most data in this field available for analysis. In 2020 (the most recent available year at time of writing), the US pet population was estimated to include 86.3 million dogs and 61.1 million cats [36]. In contrast, within all European nations combined, there were an estimated 90 million dogs and 110 million cats [37].

US pet ownership levels are regularly surveyed and reported within the American Veterinary Medical Association (AVMA) *Pet Ownership and Demographics Sourcebook* [36] and the American Pet Products Association (APPA) *National Pet Owners Survey* [38]. Due to differences in survey methods, pet ownership is consistently reported as lower within the former. For 2018, the AVMA reported a lower estimate of overall pet ownership (56.8%), than the APPA (67.0%) [39]. To ensure the most conservative estimations of the environmental impacts of pet food, for the US calculations the AVMA figures for 2020 were used in this study.

For global calculations, the global population was estimated as 471 million pet dogs, and 373 million pet cats kept worldwide in 2018 [12]. These were considered the most reliable recent, global figures, among various sources describing recent years (e.g., [40]). Hence for the global calculations, these 2018 figures were used in this study.

#### Dietary energy requirements of dogs, cats and people

First, following Okin [7], the dietary energy consumed by US dogs and cats was calculated. Maintenance energy requirements (MERs) describe “the amount of energy an animal needs to support energy equilibrium and accounts for thermoregulation, spontaneous activity and exercise. It also accounts for energy lost as heat during dietary thermogenesis or the metabolism and digestion of foods.” [41].

Domestic cats are relatively uniform in body weight (BW) reflecting their consistent roles as companion animals, notwithstanding exceptions such as cats used in laboratories, who are far fewer in number. In contrast, dog breeds vary dramatically in size from toy, small, medium, large to giant [42]. Canine MERs also vary significantly with husbandry type and activity level. These are greatest for racing dogs, followed by working and hunting dogs, and finally, pet and kennelled dogs. MERs appear equal between sexes, but are lower in neutered compared to sexually intact dogs [43]. Despite these variations, it’s nevertheless possible to determine average body weights and MERs, for both dogs and cats.

Until recently, the most accurate estimations of canine and feline calorific requirements were those supplied by the National Research Council (NRC) of the United States National Academies of Sciences, Engineering, and Medicine. According to the NRC [44], dogs’ average daily energy requirements in kJ are 544 (kg BW)^-0.75^ (where kg BW = kg of body weight), and cats’ average daily energy requirements in kJ are 418 (kg BW)^-0.67^. The NRC based its recommendations on the published studies available at the time. Since then, however, a range of additional studies have provided further evidence, particularly concerning the energy requirements of pet dogs. These may differ significantly from those of dogs kept in a kennelled environment, which commonly form the basis for controlled studies.

After systematically reviewing 29 published studies (in their final dataset), including 70 treatment groups and a total of 713 dogs, Bermingham *et al*. ([43], Table 3) found an average body weight for dogs of 20.1 kg, and an average MER of 1,351 kcal/day. Similarly, with respect to cats, after systematically reviewing 42 publications describing studies of cats (with 115 treatment groups included in their final analysis), Bermingham *et al*. ([45], Table 2) found an average body weight for domestic cats of 4.1 kg, and an average MER of 222·1 kcal/day. These figures were used in conjunction with population totals for dogs and cats, globally and within the US, to calculate total annual dietary energy requirements.

Energy requirements for average men and women were taken from the UK Government Dietary Recommendations [46], using the age bracket with the greatest energy requirements: 19–64 years of age. This yielded the maximum energy requirements of humans compared to dogs and cats, and hence, the most conservative study results. These daily energy requirements were: men– 2,500 kcal, women– 2,000 kcal. These figures were used in conjunction with population totals within the US and globally, to calculate total annual dietary energy requirements for people.

#### Total energy from animal sources (EA), consumed by dogs, cats and people

Next, utilising dietary energy proportions attributable to animal sources within pet food [7], the total dietary energy provided by animal sources was calculated for dogs and cats. For humans, the dietary energy attributable to animal sources was calculated using Food and Agriculture Organisation of the United Nations (FAO) data, for the US in 2020, and globally in 2018 [47]. These FAO data reflected food supplied rather than consumed, and the dietary proportion of animal products consumed was presumed to be equal to the dietary proportion supplied. That is, (in the absence of data to the contrary), the proportions of losses, wastage and overconsumption after supply, were assumed to be equal between the animal and non-animal dietary fractions [47]. These dietary energy proportions supplied by animal sources were then applied to the dietary energy required annually by dogs, cats and people, within the US and globally, to quantify these E_A_ and E_non-A_ fractions.

### 2. Animal-based ingredients used to feed dogs, cats and people

Determination of the natures and quantities of pet food ingredients has historically been difficult, due to variations in formulation and lack of industry transparency. In 2020 however, Decision Innovation Solutions (DIS) conducted a study examining the ingredient composition of US dog and cat diets. Their report [48] was supplemented by online data [49], providing ingredients and tonnages used within US dog and cat food from July 2018 –June 2019. These data were used to analyse ingredients used within US dog and cat food at that time.

Animal sourced-ingredients within pet food include those normally consumed by humans (human-consumable–HC), and others not normally consumed by humans (non-human-consumable–NHC). A HC example is meat, and NHC examples include animal by-products (ABPs), and their derivatives such as meat meal. After considering the ingredients used within pet food, the animal-sourced ingredients were split into HC and NHC sources. These comprised the HC and NHC dietary fractions. Each was studied separately. However, to enable subsequent calculations, dietary energy was assumed to be equally distributed across all animal-sourced ingredients. This seemed reasonable, given that the energy density of a variety of meats including poultry and fish, have all been reported to be around 200 kcal/100 g [50]. This step allowed ingredient proportions by mass to be considered proportional to dietary energy supplied.

#### Human-consumable (HC) and non-human-consumable (NHC) ingredients within dog and cat food

The process used to analyse HC and HNC dietary fractions, or ingredient sets, was similar in each case. The HC ingredient set was examined first. The largest HC ingredient groups within dog and cat food were identified, along with their consumption levels compared to other HC ingredient groups. For each of these largest HC ingredient groups, the livestock species used were identified. For each species, the average proportion (by mass) of livestock animals (i.e., carcasses) that provided these ingredients was sourced from scientific literature, to establish the efficiency of these livestock species at providing these HC ingredients. Next, averages were generated, weighted by consumption levels of these different livestock species, to create overall weighted averages for the largest HC ingredient groups within dog and cat food. This represented the proportion of ‘average’ livestock animals that provided these HC ingredients. This indicated the efficiency of providing these HC ingredients. These largest HC ingredient groups were then used as proxies for all other ingredient groups within the HC ingredient sets, for both dog and cat food. ‘Organ meats’ were excluded from the meat groups within dog and cat food, as these derived from multiple species which were not specified. This meant that the ‘organ meat’ contributions within dog and cat food were effectively assigned the same weighted averages attributed to the rest of these meat groups.

This process was then repeated for the NHC ingredient sets within dog and cat food. In the subsequent step this allowed comparison of the overall efficiencies of average livestock animals at supplying the HC and NHC ingredient sets–or dietary fractions–within dog and cat food.

### 3. Average livestock numbers (L) required to supply HC and NHC dietary fractions, for dogs, cats and people

The numbers of average livestock animals (L) required to provide animal-sourced dietary energy from HC sources (the HC fraction), is directly proportional to the magnitude of that HC dietary fraction:

$$L_{HC}=CF\;\text{x}\;HC\;fraction$$

Where CF = a conversion factor, which includes excess requirements to account over-feeding and food wastage. To facilitate calculations, these excesses were assumed to occur in the same proportions, among dogs, cats and humans.

For humans, all animal-sourced dietary energy comes solely from HC sources. Hence, the total average livestock animal numbers required to supply the animal-sourced dietary energy within human food was simply:

$$L_{humans}=(CF\;\text{x}\;HC\;fraction)$$

Within dog and cat food however, animal-sourced dietary energy is supplied by both HC and NHC sources. However, the same CF cannot be used, for both HC and NHC fractions. The proportions of average livestock animals supplying HC components, were expected to differ from the proportions suppling NHC components. As noted in the previous step, these proportions indicated the overall efficiencies of average livestock animals at supplying the HC and NHC dietary fractions within dog and cat food. The ratio of these proportions was used to create efficiency factors (EFs), which compared the efficiency of production of the HC and HNC dietary fractions. These EFs were then used to calculate the relative numbers of average livestock animals used within the diets of dogs and cats:

$$L_{dogs}=(CF\;\text{x}\;HC\;fraction)\;+(CF\;\text{x}\;NHC\;fraction\;\text{x}\;EF_{dogs})$$

$$L_{cats}=(CF\;\text{x}\;HC\;fraction)+(CF\;\text{x}\;NHC\;fraction\;\text{x}\;EF_{cats})$$

#### Proportionate livestock consumption by dogs, cats and people

Collectively, the HC and NHC fractions comprised the animal-sourced ingredients used. The relative consumption of total animal-sourced dietary energy consumed by dogs, cats and people (calculated in an initial step), was combined with the relative consumption of average livestock animals required to collectively produce the animal-sourced (*HC* + *NHC*) fractions within these diets (calculated in the prior step), to calculate the proportionate consumption levels for dogs, cats and people.

When calculating proportionate livestock use globally, global averages for NHC and HC consumption proportions within pet food ingredients were used [51] rather than relying on the US pet food ingredients [49] analysis. These global averages differed from US averages, as a significantly higher proportion of NHC ingredients are used within pet food globally, compared to US pet foods.

### 4. Effects on sustainability indicators of vegan diets for dogs, cats and people

Having enabled a proportionate attribution of the total impacts of the livestock sector, to the diets of dogs, cats and people, data for a range of environmental sustainability metrics were calculated.

#### Number of ‘food animals’ spared from slaughter

The proportionate consumption of livestock animals by dogs, cats and people was applied to the numbers of terrestrial animals killed for food in the US in 2020, and globally in 2018 [52]. Next, this was applied to the numbers of aquatic animals estimated to have been killed to maintain the U.S. food supply in 2013 [53], and within the US and globally from 2016–2017 (the years available) [54]. This enabled determination of the numbers of animals who would no longer be slaughtered annually, were nutritionally-sound vegan diets instead used to feed dogs, cats and people.

#### Various environmental impacts

As noted previously, the calories supplied by pet food are comprised of E_A_ and E_non-A_ fractions, and these proportions vary between dog and cat food. Transition to vegan pet food would result in no change in environmental impacts for the existing E_non-A_ fraction. However, impacts would change for the E_A_ fraction. To determine relative environmental impacts of animal- vs plant-based ingredients that could be consumed if dogs and cats transitioned on to vegan diets, two data sources were used.

In 2018 Poore and Nemecek provided calculations of a range of environmental impacts associated with the production of 52 plant- and animal-sourced food ingredients, using 2009–2011 averages [55]. They calculated land and water use (freshwater and stressed water–see following), GHG emissions as CO_2_ equivalents, acidifying emissions as SO_2_ equivalents, and eutrophifying emissions as PO_4_^3-^ equivalents. The components included within these are indicated in S1 Table in S1 File.

For GHGs, IPCC [56] AR5 100-year characterisation factors were used, which are the most commonly-used indicators of the impacts of GHGs on the mid- to long-term climate. These data also included direct and indirect impacts of GHGs, and climate-carbon feedbacks–the effects of climate change on factors affecting CO_2_ emission, such as the land and ocean carbon cycles, and radiative forcing. Data on the acidification and eutrophication emissions relied on CML2 baseline method characterisation factors [57]. Scarcity-weighted freshwater withdrawals relied on the WULCA consensus characterization model for water scarcity footprints (AWARE), which quantifies the relative available water remaining per area (water scarcity or stress–Str-Wt), once the demand of humans and aquatic ecosystems has been met. The resulting characterization factor from 0.1–100 indicates the potential to deprive another user (human or ecosystem) when consuming water in an area [58].

Poore and Nemecek’s data [55] quantifying the environmental impacts of these 52 plant- and animal-sourced food ingredients were examined. A small number of ingredients were excluded due to uncertainty about whether these were entirely plant- or animal-based. For dog and cat diets, ingredients unlikely to be used within animal- or plant-based diets for these species, were also excluded, and the remainder were divided into animal- and plant-based ingredients. Production volumes were supplied for all ingredients based on 2009–2011 averages, including amounts for food and food waste. Production volumes including non-food purposes were also supplied but not used, as these included uses such as biofuel and textiles (e.g., leather) production, rather than ingredients that are, or could be, consumed by dogs, cats or people.

Based on the production volumes for food and food waste, weighted averages were derived for these ingredient sets, for all of the above environmental impact categories. Ratios for the relative impacts of plant- versus animal-based ingredient consumption were then calculated (‘W’ in the following). This process was then repeated to determine the same relative environmental impacts, for human diets. In this case, the ingredients unlikely to be used within dog or cat diets, were included, as these are used within human diets.

Additionally, Reijnders and Soret [59] provided the relative impacts of meat protein production compared to plant protein production, for a range of environmental sustainability parameters. Most were superseded by the more recent Poore and Nemecek data [55], but Poore and Nemecek did not provide data for biocide use. Hence Reijnders and Soret’s ratio for biocide use was also included.

When switching to vegan pet food–i.e., replacing all animal-sourced calories, with plant-based ingredients, the impacts due to the E_A_ fraction decrease–not to 0, but to 1 –which is the relative impact if plant-based ingredients are used instead. Hence the reduction in impact through switching all animal-sourced calories to vegan ingredients (alternatively, the increase in impact accruing through use of animal-based ingredients), is:

$$(W_{j}–1)\;\text{x}\;E_{A}$$

W = livestock production impacts due to the E_A_ fraction

j = environmental impact category: land use, water use, GHG emissions, acidifying emissions, eutrophifying emissions or biocides

E_A_ = proportion of dietary energy derived from animal sources

 

The E_A_ values for dog, cat and humans diets, calculated previously, were then used to calculate these reductions in impact for all categories ‘j’. These were then added to the relative impacts of vegan diets (1) to determine total impacts associated with meat-based diets. The percentage reductions that would be achieved by replacement with vegan diets were then calculated. Finally, these percentage impact reductions within each diet, were multiplied by the proportions of total livestock consumption attributable to the diets of dogs, cats and humans respectively, both within the US and globally, to determine the reductions in total livestock sector impacts that would be expected after transitioning to vegan diets.

#### Additional people, dogs and cats who could be fed using food energy savings

As noted, the calories supplied within pet food come from two dietary fractions: E_A_ and E_non-A_. For the existing E_non-A_ fraction, no excess energy would result from transitioning to a vegan diet, as this fraction would not change. However, excess dietary energy is available within the E_A_ fraction, because most of the plant calories fed to livestock animals are used to support their bodily growth and maintenance processes, rather than directly producing consumable products [35]. After considering the average American consumption of beef, pork, poultry, other meats including fish, milk and eggs, Pimentel and Pimentel [1] reported that for every 1 kg of high-quality animal protein produced, livestock animals are fed about 6 kg of plant protein, which are produced, in turn, from many additional kg of grain and forage.

When considering the average loss-adjusted feed conversion ratio for beef+lamb, pork, and poultry, weighted by their relative availability in the diets of American people [60], Okin [7] determined that 4.7 J of plant energy were required to produce 1.0 J of meat energy. For the purposes of this study, this was generalised to all HC animal-sourced ingredients. Hence, on average 3.7 J of energy were considered to be lost during conversion from plant to HC animal-sourced ingredients. These 3.7 J of excess dietary energy could instead be freed for direct consumption as plant-sourced ingredients, when using a vegan diet.

As noted previously, the efficiencies of average livestock animals, at providing the HC and NHC dietary fractions, differed by an efficiency factor (EF), which was different for dog and cat food. For the less efficient dietary fraction, correspondingly more livestock animals were required, further reducing the efficiency of conversion from plant energy below 1.0/3.7. The differences in the numbers of livestock animal required, correspond to the EF multiples calculated previously. For example, as noted for dogs, *L*_*dogs*_ = (*CF* x *HC fraction*) + (*CF* x *NHC fraction* x *EF*_*dogs*_). Hence, for the less efficient HC or NHC dietary fraction, conversion to plant energy decreases in efficiency, by these EF same multiples. Hence, the excess dietary energy freed via direct consumption of plant ingredients, increases by these factors.

Accordingly, the excess dietary energy that would be available, were plant sources consumed directly instead of converting them to HC and NHC animal-sourced ingredients, for dog, cat and human diets, was calculated as follows. For human diets, the NHC fraction = 0.


$$\text{Dog}\;\text{food:}\;E_{A}{}_{dogs}\;\text{x}\;[HC\;+\;(NHC\;\text{x}\;EF_{dogs})]\;\text{x}\;3.7$$

$$\text{Cat}\;\text{food}:E_{A\;cats}\;\text{x}\;[HC\;+\;(NHC\;\text{x}\;EF_{cats})]\;\text{x}\;3.7$$

$$\text{Human}\;\text{food}:E_{A\;humans}\;\text{x}\;[HC\;+\;(NHC=0)]\;\text{x}\;3.7$$

These dietary food energy savings were calculated, and then compared to the annual dietary energy requirements of US people in 2020 (calculated in an earlier step), to determine the number of additional Americans who could be fed by consuming this energy directly in the form of plant-based ingredients, i.e., within a vegan diet.

These steps were repeated using E_A_ consumption for dogs, cats and humans globally. In this case global (rather than US) averages for NHC and HC consumption proportions within pet food ingredients were used as noted previously [51]. These dietary food energy savings were calculated, and then compared to the annual dietary energy requirements of all people globally in 2018, to determine the number of additional people who could be fed by consuming this energy directly, i.e., within a vegan diet.

Finally, the dietary food energy savings within dog and cat food were compared to the annual dietary energy requirements of these species, to determine the numbers of additional animals of the same species who could be fed if dogs and cats were transitioned on to nutritionally-sound vegan diets. These calculations were also performed considering both the 2020 US and 2018 global populations.

Within various data tables, data were often displayed as rounded values, e.g., rounded to one decimal place. However, where these data were used within subsequent calculations, the underlying exact values were often used, to achieve greater accuracy.

## Results

### Dietary energy requirements of dogs, cats and people

Given the 2020 US pet populations of 86.3 million dogs and 61.1 million cats [36], the total daily and annual MER requirements for dogs and cats were calculated (Table 1). In comparison, the 2020 US human population totalled 166.2 million women and 162.8 million men [61]. The average daily and annual energy requirements for US men and women are also provided in Table 1.

**Table 1:** Energy requirements of US people, dogs, and cats in 2020. After Okin [7].

Population	No. (millions)	Daily Individual Energy (kcal)	Daily Population Energy (Tcal)	Annual Population Energy (Tcal)	Annual Population Energy (PJ)
**Men**	162.8 (49.5%)	2,500	407.0	148,555.0	621.6
**Women**	166.2 (50.5%)	2,000	332.4	121,326.0	507.6
**Men + women**	**329.0 (100.0%)**				**1,129.2 (= 3.43/million people)**
**Dogs**	86.3 (58.5%)	1,351	116.6	42,555.8	178.1 (= 2.06/million dogs)
**Cats**	61.1 (41.5%)	222.1	13.6	4,953.2	20.7 (= 0.34/million cats)
**Dogs + cats**	**147.4 (100.0%)**				**198.8**

Note: Energy requirements are MERs: maintenance energy requirements. 1 kcal = 4.184 kJ.

As noted, for global calculations, the 2018 estimations of 471 million dogs, and 373 million cats kept worldwide were used [12]. In comparison, the 2018 global human population totalled 3.9 billion men and 3.8 billion women [62]. The energy requirements for these populations are similarly provided in Table 2.

**Table 2:** Energy requirements of people, dogs, and cats, globally in 2018. After Okin [7].

Population	No. (millions)	Daily Individual Energy (kcal)	Daily Population Energy (Tcal)	Annual Population Energy (Tcal)	Annual Population Energy (PJ)
**Men**	3,866 (50.3%)	2,500	9,665.0	3,527,725.0	14,760.0
**Women**	3,818 (49.7%)	2,000	7,636.0	2,787,140.0	11,661.4
**Men + women**	**7,684 (100.0%)**				**26,421.4 (= 3.44/million people)**
**Dogs**	471 (55.8%)	1,351	636.3	232,257.2	971.8 (= 2.06/million dogs)
**Cats**	373 (44.2%)	222.1	82.8	30,237.8	126.5 (= 0.34/million cats)
**Dogs + cats**	**844 (100.0%)**				**1,098.3**

Note: Energy requirements are MERs: maintenance energy requirements. 1 kcal = 4.184 kJ.

#### Total energy from animal sources (EA), consumed by dogs, cats and people

Okin [7] analysed premium dog food (n = 102), market-leading dog food (n = 9), premium cat food (n = 163), and market-leading cat food (n = 9) products within the US. He examined the mass of the five ingredients listed first within these pet foods (with each assumed to be virtually 20% by weight), and the energy density of these ingredients. He estimated the total fraction of calories derived from animal-based ingredients (E_A_) to be 34% ± 4% for dog food, and 31% ± 4% for cat food. Applying these proportions to the dietary energy required annually by dogs and cats within the US and globally, gave the E_A_ amounts in Tables 3 and 4. The remaining dietary energy was derived from non-animal sources (E_non-A_).

**Table 3:** Proportion of dietary energy attributable to animal and non-animal sources, in the diets of US dogs, cats and humans in 2020.

	E_non-A_ (PJ)	%	E_A_ (PJ)	%	E_A + non-A_ (PJ)	Total dietary E consumption (%)
**Dogs**	117.5	66.0%	60.6	34.0%	178.1	13.4%
**Cats**	14.3	69.1%	6.4	30.9%	20.7	1.6%
**Humans**	805.1	71.3%	324.1	28.7%	1,129.2	85.0%
**Total dietary E consumption**					**1,328.0**	**100.0%**
**Dogs + Cats**	131.8	66.3%	67.0	33.7%	198.8	15.0%

**Table 4:** Proportion of dietary energy attributable to animal and non-animal sources, in the diets of dogs, cats and humans globally, in 2018.

	E_non-A_ (PJ)	%	E_A_ (PJ)	%	E_A + non-A_ (PJ)	Total dietary E consumption (%)
**Dogs**	641.4	66.0%	330.4	34.0%	971.8	3.5%
**Cats**	87.4	69.1%	39.1	30.9%	126.5	0.5%
**Humans**	21,480.6	81.3%	4,940.8	18.7%	26,421.4	96.0%
**Total dietary E consumption**					**27,519.7**	**100.0%**
**Dogs + Cats**	728.8	66.4%	369.5	33.6%	1,098.3	4.0%

Considering human diets, within the US in 2020, an average of 3,926 kcal were supplied daily. 1,125 kcal (28.7%) of this came from animal produce. Globally in 2018, an average of 2,961 kcal were supplied daily, of which 553 kcal (18.7%) were from animal produce [47]. These were assumed to reflect the E_A_ proportions consumed within human diets in the US and globally. Applying these proportions to the dietary energy required annually by people resulted in the E_A_ and E_non-A_ amounts in Tables 3 and 4.

### Animal-based ingredients used to feed dogs, cats and people

In total, approximately 8.65 million tons of animal- and plant-based ingredients were included within 542 ingredients (after standardization, e.g., to eliminate duplication), that were used to produce around 9.8 million tons of US dog and cat food annually. These were sold from mid 2018–mid 2019.

Just under half of ingredients by weight within US dog and cat food were not animal-based. Non animal-based ingredients supplied 71.3% of the dietary energy consumed by US people, and 66.3% of the dietary energy jointly consumed by US dogs and cats (Table 3). After further aggregation into ingredient groups, non-animal ingredients comprised 47.6% of dog food ingredients, 44.8% of cat food ingredients and 46.9% of all dog and cat food ingredients. These included whole grains (barley, corn, oats and wheat), mill feeds (malted barley, corn gluten feed, corn meal, rice flour, etc.), soy products (soybean meal, soy protein concentrates, etc.), fruits and vegetables (dried beans, carrots, green beans, celery, tomatoes, squash, etc.), root products (peanuts, peanut butter, chicory root, etc.), vegetable oils (soybean oil, canola oil, coconut oil, etc.) and sweeteners (sugar, corn sugar, etc.).

**Fig 2:**
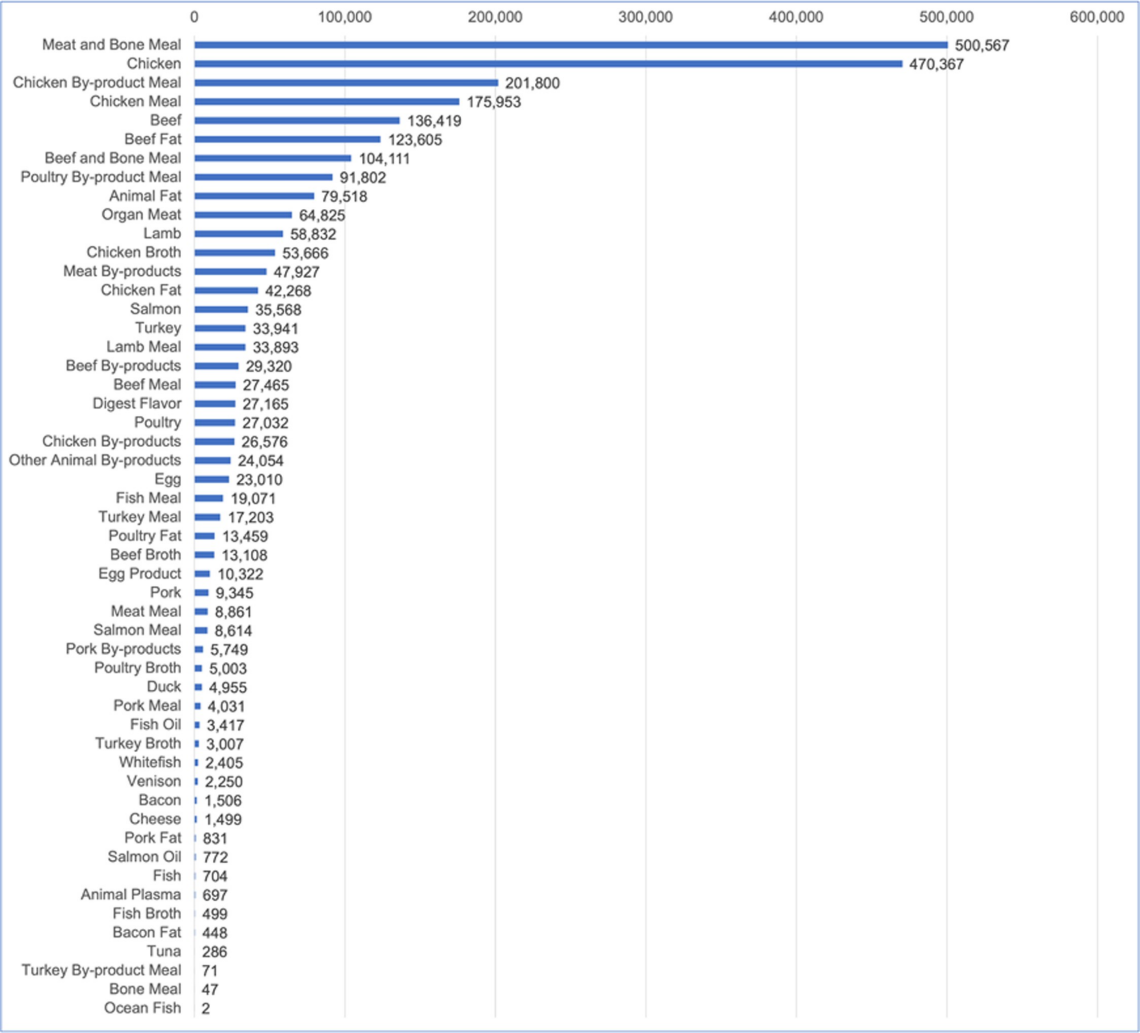
Animal-based ingredients used within US dog food from 2018–2019, in tons. Data source: [49].

**Fig 3:**
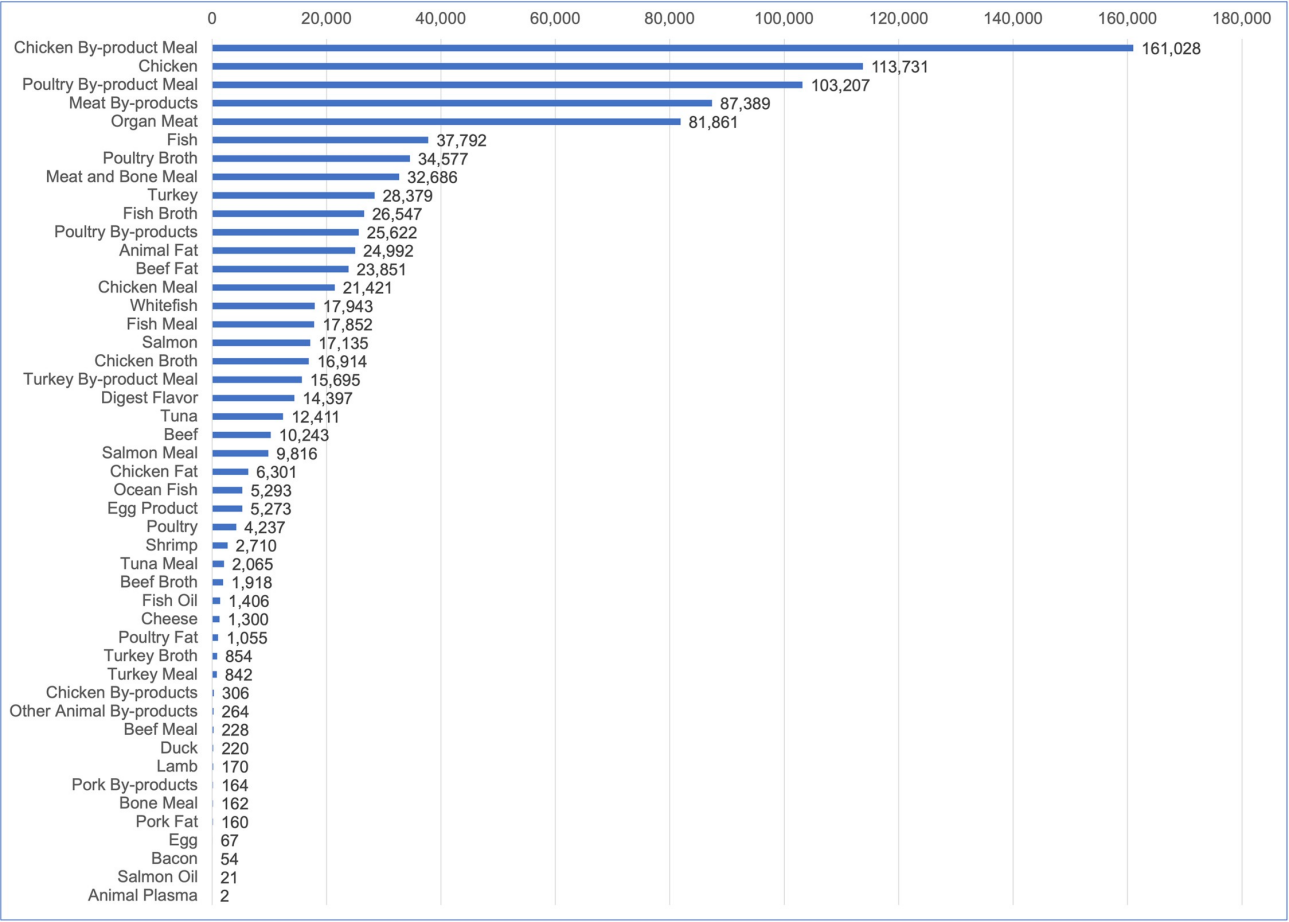
Animal-based ingredients used within US cat food from 2018–2019, in tons. Data source: DIS [49].

After aggregation into ingredient groups, 52 animal-based ingredients comprised (by mass) 52.4% of the ingredients used within dog food, 55.2% of the ingredients used within cat food, and 53.1% of all dog and cat food ingredients (Figs 2 and 3). Their main categories are provided in Table 5. HC sources included meat and fishery ingredients, fats and oils, animal broths, dairy and egg products. ‘Fishery ingredients’ included a variety of fish and fish products such as salmon, tuna, whitefish, cod, etc., fish oil products, anchovies, as well as crabs, mussels, kelp, kelp meal, algae and seaweed meal ([48], p. 26). NHC sources included animal by-products (ABPs), meat and bone meal derived from animal by-products (ABP derivatives), very small amounts of digest flavourant, and animal plasma.

**Table 5:** Animal-based ingredient categories used within US dog and cat food from 2018–2019 (tons). Data source: [49].

** Ingredient group**	**HC or NHC**	**Dog food**	**%**	**Cat food**	**%**	**Dog + Cat food**	**%**
Animal meal	NHC	1,193,490	46.3%	365,001	37.6%	1,558,491	43.9%
’Meat’ (incl. poultry, organ meats, but excl. fish)	HC	809,473	31.4%	238,895	24.6%	1,048,368	29.5%
Fats & oils	HC	264,317	10.3%	57,786	6.0%	322,103	9.1%
By-products	NHC	133,625	5.2%	113,744	11.7%	247,370	7.0%
Broth	HC	75,283	2.9%	80,811	8.3%	156,094	4.4%
Fishery ingredients	HC	38,966	1.5%	93,285	9.6%	132,251	3.7%
Other	NHC	27,861	1.1%	14,399	1.5%	42,260	1.2%
Dairy and eggs	HC	34,831	1.4%	6,639	0.7%	41,470	1.2%
**Totals**	** **	**2,577,847**	**100.0%**	**970,560**	**100.0%**	**3,548,407**	**100.0%**

Note: All ingredient groups were human-consumable (HC) other than by-products, animal meal and ‘other’ (digest flavourant and animal plasma). These were non-human consumable (NHC).

The most common animal-sourced ingredient group overall was animal meal, which comprised 43.9% of the animal-based ingredients within dog and cat food combined (Table 5). Rendered protein meals (animal meals) are produced from solid materials remaining after removal of water and fat from ABPs [63]. The three most common sources of animal meal in dog food (in order) were unspecified meat and bone, chicken, and beef and bone (S2 Table in S1 File). In cat food the three most common sources (in order) were chickens, unspecified poultry, and unspecified meat and bone (S10 Table in S1 File). Multiple other species (including aquatic) were also used.

The next most common animal-sourced ingredient group was ‘meat’ including poultry but excluding fish, which comprised 29.5% of the animal-based ingredients within dog and cat food combined (Table 5). The three most common types of meat in dog food (in order) were chicken, beef and ‘organ meat’ (S3 Table in S1 File). In cat food the three most common sources (in order) were chickens, organ meat, and turkey (S11 Table in S1 File). Organ meats were defined as including livers, hearts and lungs. Organs such as the liver, kidney, heart, brain, intestine, tongue, spleen are human-consumable and are also termed ‘variety meats’ [64].

#### Human-consumable (HC) ingredients within dog and cat food

The HC ingredient groups within dog and cat food were meat (including poultry and organ meats), fats and oils, broth, fishery ingredients, and dairy and eggs (Table 5). Of these, meat was the largest group. For dog food, HC ingredients comprised 47.4% of all animal-sourced ingredients, and meat comprised 66.2% of this HC group. For cat food, HC ingredients comprised 49.2% of all animal-sourced ingredients, and meat comprised 50.0% of this HC group.

The meat used within US pet food was chicken, organ meat, beef, turkey, lamb, poultry, pork, duck, venison and bacon. The proportions normally derived from carcasses of the source species, and their levels of inclusion within dog and cat food, are given in Tables 6 and 7. The proportionate use of these species differed between dog and cat food, resulting in different weighted averages for meat per average carcass. For dog and cat food respectively, these weighted averages were 53.0% and 58.8%. As noted, meat was the largest ingredient group among all HC ingredients used within dog and cat food, and these meat weighted averages were used as proxies for all HC ingredients within these diets.

**Table 6:** Meat proportions within carcasses of animal species used within dog food.

**Species**	**Annual consumption**		**Meat per carcass (%)**
	**tons**	**%**	
Chicken	470,367	63.2%	59.9%
Beef	136,419	18.3%	39.1%
Lamb	58,832	7.9%	23.2%
Turkey	33,941	4.6%	61.5%
Poultry (unspecified)	27,032	3.6%	60.0%
Pig (pork + bacon)	10,851	1.5%	47.1%
Duck	4,955	0.7%	60.0%
Venison	2,250	0.3%	25.6%
**Totals**	**744,648**	**100.0%**	** **
**Weighted average**	** **	** **	**53.0%**

Note: 64,825 tons of ’organ meat’ was sourced from multiple species annually, and was excluded. Carcasses were live weights at slaughter. For ducks, Pekin ducks were used. Poultry percentages were defined as the weighted average of chicken + turkey + duck. Venison yields were based on fast-growing red deer stags. Due to differences in sources used, in some cases meat + ABPs > 100% for the same species.

**Table 7:** Meat proportions within carcasses of animal species used within cat food.

**Species**	**Annual consumption**		**Meat per carcass (%)**
	**tons**	**%**	** **
Chicken	113,731	72.4%	59.9%
Turkey	28,379	18.1%	61.5%
Beef	10,243	6.5%	39.1%
Poultry (unspecified)	4,237	2.7%	60.2%
Duck	220	0.1%	60.0%
Lamb	170	0.1%	23.2%
Pig (bacon)	54	0.0%	47.1%
**Totals**	**157,034**	**100.0%**	** **
**Weighted average**	** **	** **	**58.8%**

Note: 81,861 tons of ’organ meat’ was sourced from multiple species annually, and was excluded. Carcasses were live weights at slaughter. For ducks, Pekin ducks were used. Poultry percentages were defined as the weighted average of chicken + turkey + duck. Due to differences in sources used, in some cases meat + ABPs > 100% for the same species.

#### Non-human-consumable (NHC) ingredients within dog and cat food

The NHC ingredient groups within dog and cat food were animal meal (which is derived from ABPs), ABPs and ‘other’ (digest flavourant and animal plasma) (Table 5). Of these, animal meal was the largest group. For dog food, NHC ingredients comprised 52.6% of all animal-sourced ingredients, and animal meal comprised 88.1% of this NHC group. For cat food, NHC ingredients comprised 50.8% of all animal-sourced ingredients, and animal meal comprised 74.0% of this NHC group.

The meat meal was derived from ABPs of production of the following meats: unspecified (meat and bone), chicken, unspecified (poultry), beef and bone, unspecified (fish), lamb, salmon, turkey, pork and tuna. The proportions normally derived from carcasses of the source species, and their levels of inclusion within dog and cat food, are given in Tables 8 and 9. The proportionate use of these species differed between dog and cat food, resulting in different weighted averages for ABPs per average livestock carcass. For dog and cat food respectively, these weighted averages were 39.2% and 31.3%. As noted, animal meal was the largest ingredient group among all NHC ingredients used within dog and cat food, and these weighted averages were used as proxies for all NHC ingredients within these diets.

**Table 8:** Meat meals used as ingredients within dog food.

Meal type	Annual consumption		ABPs per carcass (%)
	tons	%	
Meat and bone meal	509,476	42.7%	39.2%
Chicken meal	377,753	31.7%	29.2%
Beef and bone meal	131,576	11.0%	66.0%
Poultry by-product meal	91,802	7.7%	29.2%
Lamb meal	33,893	2.8%	68.0%
Fish meal	19,071	1.6%	45.0%
Turkey meal	17,274	1.4%	36.4%
Salmon meal	8,614	0.7%	45.0%
Pork meal	4,031	0.3%	52.0%
**Totals**	**1,193,490**	**100.0%**	
**Weighted average**			**39.2%**

Note: ’Meat and bone’ meal refers to unspecified species, and included ’meat meal’ and ’bone meal’. For this, the weighted average of all other species was used. ’Chicken meal’ included ’chicken by-product meal’. ’Beef and bone’ meal included ’beef meal’. ’Turkey meal’ included ’turkey by-product meal’. For unspecified ’poultry by-product meal’, ’chicken meal’ was used. For unspecified ’fish meal’, ’salmon meal’ was used. Due to differences in sources used, in some cases meat + ABPs > 100% for the same species.

**Table 9:** Meat meals used as ingredients within cat food.

Meal type	Annual consumption		ABPs per carcass (%)
	tons	%	
Chicken meal	182,449	50.0%	29.2%
Poultry meal	103,207	28.3%	29.8%
Meat and bone meal	32,847	9.0%	31.3%
Fish meal	17,852	4.9%	45.9%
Turkey meal	16,536	4.5%	36.4%
Salmon meal	9,816	2.7%	45.0%
Tuna meal	2,065	0.6%	50.0%
Beef meal	228	0.1%	66.0%
**Totals**	**365,001**	**100.0%**	
**Weighted average**			**31.3%**

Note: ’Meat and bone’ meal refers to unspecified species, and included ’bone meal’. For this, the weighted average of all other species was used. ’Chicken meal’ included ’chicken by-product meal’. ’Turkey meal’ included ’turkey by-product meal’. For unspecified ’poultry by-product meal’, the weighted average of the other poultry species (chicken and turkey) was used. For unspecified ’fish meal’, the weighted average of the other fish species (salmon and tuna) was used. Due to differences in sources used, in some cases meat + ABPs > 100% for the same species.

### Average livestock numbers (L) required to supply HC and NHC dietary fractions, for dogs, cats and people

As noted, the total numbers of average livestock animals required to supply the E_A_ within human food were:

$$L_{humans}\;=\;CF\;\text{x}\;HC\;fraction$$

For dogs, NHC sources were, on average, 39.2%/53.0% = 0.740 times as efficient compared to HC sources. They required 1/0.740 = 1.352 times the number of average livestock animals to provide the same ingredient mass. Hence, the total average livestock animal numbers required to supply the animal-sourced dietary energy within dog food were:

$$L_{dogs}\;=\;(CF\;\text{x}\;HC\;fraction)\;+\;(CF\;\text{x}\;NHC\;fraction\;\text{x}\;1.352)$$

Similarly for cats, NHC sources were, on average, 31.3%/58.8% = 0.532 times as efficient compared to HC sources. They required 1/0.532 = 1.879 times the number of average livestock animals. Hence, the total average livestock animal numbers required to supply the animal-sourced dietary energy within cat food were:

$$L_{cats}\;=\;(CF\;\text{x}\;HC\;fraction)\;+\;(CF\;\text{x}\;NHC\;fraction\;\text{x}\;1.879)$$

#### Proportionate livestock consumption by dogs, cats and people, within the US in 2020

The E_A_ dietary fractions required by dogs (60.6 PJ), cats (6.4 PJ) and humans (324.1 PJ) in the US in 2020 were given in Table 3. For humans, as noted all of these animal-sourced ingredients were HC. Hence, the total number of average livestock animals required to supply the animal-sourced dietary energy within these human diets was:

$$L_{humans}\;=\;CF\;\text{x}\;324.1$$

For dog food, the E_A_ dietary fraction was comprised of (HC: 47.4% = 28.7 PJ) + (NHC: 52.6% = 31.9 PJ) = 60.6 PJ. Hence, the total number of average livestock animals required to supply the animal-sourced dietary energy within dog food was:

$$L_{dogs}\;=\;(CF\;\text{x}\;28.7)\;+\;(CF\;\text{x}\;31.9\;\text{x}\;1.352)=CF\;\text{x}\;71.8$$

For cat food, the E_A_ dietary fraction was comprised of (HC: 49.2% = 3.1 PJ) + (NHC: 50.8% = 3.3 PJ) = 6.4 PJ. Hence, the total number of average livestock animals required to supply the animal-sourced dietary energy within cat food was:

$$L_{cats}\;=(CF\;\text{x}\;3.1)\;+\;(CF\;\text{x}\;3.3\;\text{x}\;1.879)=CF\;\text{x}\;9.3$$

Hence, the consumption of average livestock animals to supply the animal-sourced dietary energy required by US dogs, cats and humans in 2020, was 17.7% for dogs, 2.3% for cats, 80.0% for humans, and 20.0% for dogs and cats jointly (Table 10).

**Table 10:** Proportionate use of average livestock animals required to meet animal-sourced dietary energy needs, within US dog, cat and human diets in 2020.

	Livestock animals	%
**Humans**	CF x 324.1	80.0%
**Dogs**	CF x 71.8	17.7%
**Cats**	CF x 9.3	2.3%
**Total**	**CF x 405.2**	**100.0%**
**Dogs + cats**	CF x 81.1	20.0%

#### Proportionate livestock consumption by dogs, cats and people, globally in 2018

Similarly, the E_A_ dietary fractions required by dogs (330.4 PJ), cats (39.1 PJ) and humans (4,940.8 PJ) globally in 2018 were given in Table 4. For humans, as noted, all of these animal-sourced ingredients were HC. Hence, the total number of average livestock animals required to supply the animal-sourced dietary energy within these human diets was:

$$L_{humans}\;=\;CF\;\text{x}\;4,940.8$$

As noted, for US dog and cat food, NHC components comprised 52.6% and 50.8% of all animal-sourced ingredients, respectively. In comparison, the global consumption of meat meal, ABP meal and animal digest within pet food (comprising all NHC ingredients) in 2019 (the closest available year to 2018), comprised 16,416.3 kT, or 74.9% of the 21,904.5 kT total meat and meat products consumed within pet food (T = US ton) [51]. Separate figures for dog and cat food were not available; hence this 74.9% average was applied equally to dog and cat food consumed globally in 2018.

For dog food, the E_A_ dietary fraction was comprised of (HC: 25.1% = 82.9 PJ) + (NHC: 74.9% = 247.5 PJ) = 330.4 PJ. Hence, the total number of average livestock animals required to supply the animal-sourced dietary energy within dog food was:

$$L_{dogs}\;=\;(CF\;\text{x}\;82.9)\;+\;(CF\;\text{x}\;247.5\;\text{x}\;1.352)\;=\;CF\;\text{x}\;417.5$$

For cat food, the E_A_ dietary fraction was comprised of (HC: 25.1% = 9.8 PJ) + (NHC: 74.9% = 29.3 PJ) = 39.1 PJ. Hence, the total number of average livestock animals required to supply the animal-sourced dietary energy within cat food was:

$$L_{cats}\;=\;(CF\;\text{x}\;9.8)\;+\;(CF\;\text{x}\;29.3\;\text{x}\;1.879)\;=\;CF\;\text{x}\;64.8$$

Hence, the consumption of average livestock animals to supply the animal-sourced dietary energy required by dogs, cats and humans globally in 2018, was 7.7% by dogs, 1.2% by cats, 91.1% by humans and 8.9% by dogs and cats jointly (Table 11).

**Table 11:** Proportionate use of average livestock animals required to meet animal-sourced dietary energy needs, within dog, cat and human diets globally in 2018.

	Livestock animals	%
**Humans**	CF x 4,940.8	91.1%
**Dogs**	CF x 417.5	7.7%
**Cats**	CF x 64.8	1.2%
**Total**	**CF x 5,423.1**	**100.0%**
**Dogs + cats**	CF x 482.3	8.9%

### Effects on sustainability indicators of vegan diets for dogs, cats and people

The environmental impacts created by livestock animals are directly proportional to the numbers consumed within conventional (meat-based) diets. Hence, the proportions of livestock sector environmental impacts, due to following conventional diets, were, in the US in 2020: dog food– 17.7%, cat food– 2.3%, dog and cat food– 20.0%, and human food– 80.0%. Globally in 2018 these were: dog food– 7.7%, cat food– 1.2%, dog and cat food– 8.9%, and human food– 91.1%. In contrast, nutritionally-sound vegan diets, would provide a range of sustainability benefits.

#### Number of ‘food animals’ spared from slaughter

*Terrestrial animals*. Transition to nutritionally-sound vegan diets would no longer require the slaughter of livestock animals for food. Given the proportionate consumption of average livestock animals within the diets of dogs, cats and humans, this would spare billions of terrestrial animals from slaughter annually, within the US and globally (Tables 12 and 13).

**Table 12:** Terrestrial animals killed for food in 2020, within the US, used within the diets of dogs, cats and humans. Totals were sourced from FAOSTAT [52].

	**US total (2020)**	**Humans (80.0%)**	**Dogs (17.7%)**	**Cats (2.3%)**	**Dogs and cats (20.0%)**
Poultry	9,592,147,000	7,673,717,600	1,697,810,019	220,619,381	1,918,429,400
Pigs	131,639,000	105,311,200	23,300,103	3,027,697	26,327,800
Bovine animals	33,366,100	26,692,880	5,905,800	767,420	6,673,220
Sheep and goats	2,942,800	2,354,240	520,876	67,684	588,560
Other land animals	77,594	62,075	13,734	1,785	15,519
**Totals**	**9,760,172,494**	**7,808,137,995**	**1,727,550,531**	**224,483,967**	**1,952,034,499**

**Table 13:** Terrestrial animals killed for food in 2018, globally, used within the diets of dogs, cats and humans. Totals were sourced from FAOSTAT [52].

	**World total (2018)**	**Humans (91.1%)**	**Dogs (7.7%)**	**Cats (1.2%)**	**Dogs and cats (8.9%)**
Poultry	74,640,136,000	67,997,163,896	5,747,290,472	895,681,632	6,642,972,104
Pigs	1,478,059,606	1,346,512,301	113,810,590	17,736,715	131,547,305
Sheep and goats	1,047,391,220	954,173,401	80,649,124	12,568,695	93,217,819
Other land animals	726,797,375	662,112,409	55,963,398	8,721,569	64,684,966
Bovine animals	353,868,375	322,374,090	27,247,865	4,246,421	31,494,285
**Totals**	**78,246,252,576**	**71,282,336,097**	**6,024,961,448**	**938,955,031**	**6,963,916,479**

*Aquatic animals*. Aquatic animal deaths are challenging to calculate because their numbers are provided as tonnages. Harish [53] calculated the numbers of finned fish, shellfish, ‘feedfish’ (used within animal feed, primarily for livestock animals), and bycatch aquatic animals (killed within capture fisheries), that were collectively killed to maintain the U.S. food supply in 2013 (Table 14). Total U.S. fish landings reportedly remained consistent at these levels, at least through 2018. Using FAO and other sources, Fishcount.org provided similar data globally, per nation and per species (Tables 15 and 16). As demonstrated by Harish [53] (Table 14), vast numbers of ‘feedfish’ and bycatch aquatic animals were not reflected within fisheries, aquaculture and decapod numbers (Table 15).

**Table 14:** Aquatic animals killed for food in 2013, within the diets of US dogs, cats and humans (billions). Data: Harish [53].

	**US total (2013)**
‘Feedfish’	45.5 - 92.3
Shellfish	43.1
Bycatch aquatic animals	14.5 - 32.8
Finned fish	3.8
**Total**	**106.9 - 172.0**

**Table 15:** Fish and decapods consumed annually within the diets of US dogs, cats and humans (billions). Data: fishcount.org [54].

	US total
Fish ‐ fisheries (2007 ‐ 2016 avg.)	6.287 ‐ 13.512
Fish ‐ aquaculture (2017)	0.244 ‐ 0.583
Decapods (2017)	2.053 ‐ 3.336

Note: Includes all fish species with an Estimated Mean Weight (EMW), comprising 96% of total fisheries capture, 98% of aquaculture production, and 100% of decapods. Decapods were crabs and lobsters (97%), and shrimps and prawns (3%). These percentages were all based on production tonnages.

**Table 16:** Fish and decapods consumed annually within the diets of dogs, cats and humans, globally (billions). Data: fishcount.org [54].

	**World total**
Fish - fisheries (2007 - 2016 avg.)	787.458 - 2,328.767
Decapods (2017)	255.227 - 604.731
Fish - aquaculture (2017)	51.107 - 167.476

The proportions of aquatic species used within US dog and cat food respectively were 2.8% and 15.6% by mass (combining HC and NHC aquatic species and excluding animal-sourced ingredients from unspecified species) [49]. Because actual consumption levels were determined by E_A_ and by carcass provision of HC:NHC components–and because the latter were not 1:1, true consumption levels cannot be directly discerned from these 2.8% and 15.6% proportions. The proportion of overall consumption would also depend on human consumption levels. Nevertheless, if in excess of just 1% of overall consumption–as appears likely, this would equate to billions of aquatic animals being consumed within dog and cat food annually, in the US alone.

#### Various environmental impacts

As described within the Methodology, data on plant- and animal-sourced food ingredients provided by Poore and Nemecek [55] were examined. ‘Oils misc.’ and ‘sweeteners and honey’ were excluded due to uncertainty about whether these were entirely plant- or animal-based. Collectively these totalled only 0.7% by weight of these 52 ingredients.

When considering dog or cat diets, eight plant- and three animal-based ingredients or ingredient groups were excluded from further analysis as they were unlikely to be used within these diets (after considering the ingredients used within dog and cat diets [49]; e.g., sweeteners and spices (S18 Table in S1 File).

Weighted averages (based on production volumes) for the remaining 29 plant- and 12 animal-based ingredients were calculated, for each of the environmental impacts calculated by Poore and Nemecek [55]: land and water use, GHG emissions as CO_2_ equivalents, acidifying emissions as SO_2_ equivalents, and eutrophifying emissions as PO_4_^3-^ equivalents (S19 Table in S1 File). The ratios for the relative impacts of plant- versus animal-based ingredient consumption, are provided in S20 Table in S1 File. For example, for land use, the relative impact (W) of an animal-based:vegan diet = 18.911:1. As described in the Methodology, Reijnders and Soret’s ratio for biocide use [59] is also included in S20 Table in S1 File.

As noted, when considering human diets, the ingredients unlikely to be used within dog or cat diets (S18 Table in S1 File–‘Excluded’), were included, as these are used within human diets. The corresponding environmental impacts for human diets, are also provided in S20 Table in S1 File.

The relative environmental impacts in all categories (other than biocides–which did not rely on these ingredient calculations) were markedly higher for dog and cat food, compared to human diets (S20 Table in S1 File: ‘Relative impact: dog or cat (W)/human (W)’). And yet, these calculations did not account for different consumption levels of ingredients between dog, cat and human diets (other than exclusion of certain ingredients from dog and cat food, as noted in S18 Table in S1 File). Hence, this significantly underestimates the true differences, because a higher proportion of dog and cat diets (34.0% and 30.9% of calories respectively), are supplied by animal sources (which have greater environmental impacts), compared to human diets (18.7 or 28.7% of calories, globally or in the US, respectively) (Tables 3 and 4). Hence the relative impacts of dog and cat diets, compared to human diets, are actually significantly greater than indicated in S20 Table in S1 File.

Nevertheless, using those very conservative relative impacts, along with E_A_ values for dog, cat and human diets (Tables 3 and 4), the impact reductions within each diet, achievable through transition to vegan diets, were calculated as described in the Methodology. For dogs + cats, the US figure of 0.337 (33.7% in Table 3) was used rather than the global figure of 0.336 (33.6% in Table 4), as the underlying data for US pet food consumption was most likely to be accurate. For dogs and cats considered individually, the US and global figures were identical (dogs: 34.0%, cats: 30.9%, in Tables 3 and 4). These impact reductions were therefore:

$$\text{Dogs}\;:\;(W_{j}–1)\;\text{x}\;E_{A}\;=\;(W_{j}–1)\;\text{x}\;0.340$$

$$\text{Cats}\;:\;(W_{j}–1)\;\text{x}\;E_{A}\;=\;(W_{j}–1)\;\text{x}\;0.309$$

$$\text{Dogs}\;\text{+}\;\text{cats}:\;(W_{j}–1)\;\text{x}\;E_{A}\;=\;(W_{j}–1)\;\text{x}\;0.337$$

$$\text{Humans}\;(\text{US}):\;(W_{j}–1)\;\text{x}\;E_{A}\;=\;(W_{j}–1)\;\text{x}\;0.287$$

$$\text{Humans}\;(\text{global})\;:\;\;(W_{j}–1)\;\text{x}\;E_{A}\;=\;(W_{j}–1)\;\text{x}\;0.187$$

These impact reductions are also provided in S20 Table in S1 File. For example, for biocides, the additional impact within the dog food diet, accrued by animal-based ingredients, is (6.000–1) x 0.340 = 1.700, compared to a vegan diet with an impact of 1, creating a total impact for meat-based dog food of 2.700. The reduction of biocide impacts achieved via vegan dog food is 1.700/2.700 = 63.0%.

Given these impact reductions associated with vegan diets, and considering the relative proportions of livestock consumption required to supply the E_A_ within the diets of dogs, cats and humans (Tables 10 and 11), the reductions in total livestock sector impacts in each category, achieved through use of vegan diets, were calculated for the US (2020 consumption levels) and globally (2018 consumption levels) (Tables 17 and 18).

**Table 17:** Reductions in total livestock sector impacts within the US, achieved through use of vegan diets for dogs, cats or humans, based on 2020 consumption levels.

Diet	Parameter	Land Use (m^2^)	Freshwater (L)	Str-Wt WU (L eq)	GHG (kg CO_2_eq, IPCC 2013)	Acid. (kg SO_2_eq)	Eutr. (kg PO_4_^3-^eq)	Biocides
**Dog food**	Reduction of diet impact due to vegan diet	85.9%	32.7%	31.2%	75.1%	74.6%	74.7%	63.0%
	Proportion of total livestock consumption	17.7%	17.7%	17.7%	17.7%	17.7%	17.7%	17.7%
	**Reduction of total livestock impact due to vegan diet**	**15.2%**	**5.8%**	**5.5%**	**13.3%**	**13.2%**	**13.2%**	**11.1%**
**Cat food**	Reduction of diet impact due to vegan diet	84.7%	30.7%	29.2%	73.3%	72.8%	72.9%	60.7%
	Proportion of total livestock consumption	2.3%	2.3%	2.3%	2.3%	2.3%	2.3%	2.3%
	**Reduction of total livestock impact due to vegan diet**	**1.9%**	**0.7%**	**0.7%**	**1.7%**	**1.7%**	**1.7%**	**1.4%**
**Dog food + cat food**	Reduction of diet impact due to vegan diet	85.8%	32.5%	31.0%	75.0%	74.4%	74.6%	62.8%
	Proportion of total livestock consumption	20.0%	20.0%	20.0%	20.0%	20.0%	20.0%	20.0%
	**Reduction of total livestock impact due to vegan diet**	**17.2%**	**6.5%**	**6.2%**	**15.0%**	**14.9%**	**14.9%**	**12.6%**
**Human diet (US)**	Reduction of diet impact due to vegan diet	75.3%	21.4%	20.1%	56.9%	57.7%	55.8%	58.9%
	Proportion of total livestock consumption	80.0%	80.0%	80.0%	80.0%	80.0%	80.0%	80.0%
	**Reduction of total livestock impact due to vegan diet**	**60.3%**	**17.1%**	**16.1%**	**45.5%**	**46.1%**	**44.6%**	**47.1%**

**Table 18:** Reductions in total livestock sector impacts globally, achieved through use of vegan diets for dogs, cats or humans, based on 2018 consumption levels.

Diet	Parameter	Land Use (m^2^)	Freshwater (L)	Str-Wt WU (L eq)	GHG (kg CO_2_eq, IPCC 2013)	Acid. (kg SO_2_eq)	Eutr. (kg PO_4_^3-^eq)	Biocides
**Dog food**	Reduction of diet impact due to vegan diet	85.9%	32.7%	31.2%	75.1%	74.6%	74.7%	63.0%
	Proportion of total livestock consumption	7.7%	7.7%	7.7%	7.7%	7.7%	7.7%	7.7%
	**Reduction of total livestock impact due to vegan diet**	**6.6%**	**2.5%**	**2.4%**	**5.8%**	**5.7%**	**5.8%**	**4.8%**
**Cat food**	Reduction of diet impact due to vegan diet	84.7%	30.7%	29.2%	73.3%	72.8%	72.9%	60.7%
	Proportion of total livestock consumption	1.2%	1.2%	1.2%	1.2%	1.2%	1.2%	1.2%
	**Reduction of total livestock impact due to vegan diet**	**1.0%**	**0.4%**	**0.4%**	**0.9%**	**0.9%**	**0.9%**	**0.7%**
**Dog food + cat food**	Reduction of diet impact due to vegan diet	85.8%	32.5%	31.0%	75.0%	74.4%	74.6%	62.8%
	Proportion of total livestock consumption	8.9%	8.9%	8.9%	8.9%	8.9%	8.9%	8.9%
	**Reduction of total livestock impact due to vegan diet**	**7.6%**	**2.9%**	**2.8%**	**6.7%**	**6.6%**	**6.6%**	**5.6%**
**Human diet (global)**	Reduction of diet impact due to vegan diet	66.6%	15.1%	14.1%	46.2%	47.0%	45.1%	48.3%
	Proportion of total livestock consumption	91.1%	91.1%	91.1%	91.1%	91.1%	91.1%	91.1%
	**Reduction of total livestock impact due to vegan diet**	**60.6%**	**13.7%**	**12.8%**	**42.1%**	**42.8%**	**41.1%**	**44.0%**

The proportions above can be applied to a range of livestock sector impacts, to illustrate the benefits likely to accrue from transitions to vegan diets for dogs, cats and people. Examples follow for land and freshwater use, and GHG emissions.

*Land use*. In 2006, Steinfeld *et al*. [2] noted that 78% of the world’s agricultural land, and 33% of the world’s cropland, is used for livestock production. Since then, livestock numbers have increased significantly. Hence, Poore and Nemecek [5] calculated that meat, aquaculture, eggs and dairy production utilised around 83% of the world’s agricultural land. The more conservative 2006 figures alone, indicate that livestock grazing and feed crop production uses 3.9 billion ha (hectares) of land, or 30% of the non-polar terrestrial surface of the Earth. Hence, considering global consumption levels, at least the following land savings would result from vegan diets (in billion ha): dogs– 0.26 (larger than Saudi Arabia or Mexico), cats– 0.04 (larger than countries such as Japan or Germany), dogs and cats– 0.30 (larger than Argentina), humans– 2.36 (larger than Russia–the world’s largest country–combined with India) [65].

Additionally, livestock are often major sources of pollution, releasing large quantities of organic matter, pathogens and drug residues on to soil and in to rivers, lakes and coastal zones [66–68]. The 100+ million cattle produced in the US annually each generate an average of 9,000 kg of solid waste per year [67]. Livestock impacts landscapes, often profoundly diminishing biodiversity. The Amazon rainforest is among the world’s most biodiverse ecosystems. Around 70% of the previously forested Amazonian land has been converted to pastures, with much of the remaining 30% converted to croplands, largely for livestock feed [2]. Vegan diets would free up vast amounts of land, allowing rewilding and biodiversity recovery.

*Freshwater use*. The water used by the livestock sector exceeds 8% of global human water use [69]. Global animal production requires about 2,422 Gm^3^ of water per year (87.2% green, 6.2% blue, and 6.6% grey water). The green water footprint derives from precipitation. Blue water is sourced from surface or groundwater, and grey water is fresh water required to assimilate pollutants to meet water quality standards. One third of this volume is consumed by the beef cattle sector, and another 19% by the dairy sector. Almost all (98%) of water consumed is required to grow feed crops. Drinking water for the animals, service water and water for feed mixing, require only 1.1%, 0.8% and 0.03% of these water volumes, respectively [70]. Freshwater is encapsulated by the blue and grey water components. Globally, this freshwater used for animal production comprises 310.01 Gm^3^. Hence, considering global consumption levels, freshwater use reductions achieved by vegan diets would be (in Gm^3^): dogs– 7.75 (greater than all renewable water in Denmark), cats– 1.24 (greater than all renewable water in Jordan), dogs and cats– 8.99 (greater than all renewable water in Gambia), and humans– 42.47 (greater than all renewable water in Cuba) [71–73].

*Greenhouse gases*. Anthropogenic GHGs created by the livestock sector are second only to those created by the energy sector [69]. Livestock-associated GHGs come from deforestation for pasture and feed crops, pasture degradation, and from direct emissions from livestock and their waste products.

The main GHG emissions associated with livestock production are CO_2_, CH_4_, and N_2_O. Of these, 19% of CH_4_ emissions come from the livestock sector. Enteric fermentation and manure collectively contribute 80% of the methane emissions [74, 75]. Of next greatest importance is N_2_O. Livestock production contributes 15% of N_2_O emissions. Finally, livestock production contributes 1.35% of CO_2_ emissions [76]. The global warming potential of these gases varies greatly. The IPCC [56] reported a warming potential for CH_4_ of 34 CO_2_-eq, and for N_2_O of 310 CO_2_-eq, over a 100 year timeframe. The equivalent figures reported by the UNFCCC [77] for CH_4_ were 21 CO_2_-eq, and for N_2_O were (also) 310 CO_2_-eq.

The food system results in 35% of all GHGs globally, and 57% of all food sector emissions come from livestock, resulting in a total of 20% of all GHGs–or 9.8 Gt CO_2_-eq–from livestock [3]. Hence, reductions in total anthropogenic GHGs achieved by vegan diets globally would be 20% of the reductions shown in Table 18, given that Table 18 relates only to those impacts attributable to the livestock sector. As percentages of all anthropogenic GHGs, these would represent reductions of: dogs– 1.2%, cats– 0.2%, dogs and cats– 1.3%, and humans– 8.4%.

Considering the 9.8 Gt CO_2_-eq from livestock, and the reductions achieved by vegan diets shown in Table 18, these would equate to GHG emissions savings, in Gt CO_2_-eq, of: dogs– 0.57 (greater than all emissions from South Africa or the UK), cats– 0.09 (greater than all emissions from Israel or New Zealand), dogs and cats– 0.66 (greater than all emissions from Saudi Arabia or Australia), and humans– 4.13 (greater than all emissions from India or the entire EU). These refer to the total GHG emissions used for the production of all goods and services in these nations or regions, based on 2018 figures [78].

#### Additional people who could be fed using food energy savings

*Within the US in 2020*. As noted in the Methodology, an average of 4.7 J of plant energy were required to produce 1.0 J of energy from HC animal-sourced ingredients. The remaining 3.7 J of dietary energy was lost during conversion from plant- to animal-based ingredients. Within a vegan diet, this lost energy would have been available for direct consumption in the form of plant-based ingredients.

Furthermore, as noted previously, just over half of the animal-sourced ingredients within dog and cat food were supplied by NHC components. For dog food this proportion was 52.6%, and for cat food it was 50.8%. As calculated previously, the numbers of livestock animals required to provide the animal-sourced NHC fractions for dogs and cats, were respectively 1.352 and 1.879 times the numbers required to provide equivalent dietary energy as HC components. Hence, for the NHC dietary fraction, conversion to plant energy decreased in efficiency by these factors, and the excess dietary energy potentially freed via direct consumption of plant ingredients, would have increased by these factors.

Accordingly, the excess dietary energy that would be available, were plant sources used instead of all HC and NHC animal-sourced ingredients, for dog, cat and human diets within the US in 2020, would be as follows. The E_A_ quantities were provided in Table 3. For human diets, the NHC fraction = 0.


$$\begin{array}{l} \text{Dog}\;\text{food}\;:\;EA_{dogs}\;\text{x}\;[HC\;+\;(NHC\;\text{x}\;1.352)]\;\text{x}\;3.7=60.6\;PJ\;\\ \text{x}\;[47.4\%\;+\;(52.6\%\;\text{x}\;1.352)]\;\text{x}\;3.7\;=\;265.7\;PJ. \end{array}$$

$$\begin{array}{l} \text{Cat}\;\text{food}\;:\;EA_{cats}\;\text{x}\;[HC\;+\;(NHC\;\text{x}\;1.879)]\;\text{x}\;3.7\;\\ =\;6.4\;\;PJ\;\text{x}\;[49.2\%\;+\;(50.8\%\;\text{x}\;1.879)]\;\text{x}\;3.7\;=\;34.3\;PJ. \end{array}$$

Dogandcatfood:265.7PJ+34.3PJ=300.0PJ.

$$\begin{array}{l} \text{Human}\;\text{food}\;:\;EA_{humans}\;\text{x}\;[HC\;+\;(NHC\;=\;0)]\;\text{x}\;3.7\\ =324.1\;PJ\;\text{x}\;[100\%+(0)]\;\text{x}\;3.7=1,199.2\;PJ. \end{array}$$

Given this excess dietary energy, and recalling that a million US people can be fed per 3.43 PJ of dietary energy (Table 1), the numbers of additional people that could be fed through consuming this energy directly in the form of plant-based ingredients (i.e., within a vegan diet), are provided in Table 19.

**Table 19:** Proportion of the 2020 US human population who could be fed with food energy savings associated with vegan diets.

Vegan diet	Food energy savings (PJ)	People fed (millions)	% of 2020 US population
**Dog food**	265.7	77.5	23.5
**Cat food**	34.3	10.0	3.0
**Dog + cat food**	300.0	87.5	26.6
**Human food**	1,199.2	349.6	106.3

Hence, compared to using vegan diets to feed American people, the use of nutritionally-sound vegan dog food would free sufficient food energy to feed 0.22 times as many Americans. Nutritionally-sound vegan cat food would free sufficient food energy to feed 0.03 times as many, and use of vegan dog and cat food combined would free sufficient food energy to feed 0.25 times as many Americans–i.e., one quarter of the number of Americans who could be fed using the food energy saved, if all American people transitioned on to vegan diets.

*Globally in 2018*. Considering dog and cat food globally in 2018, as noted previously the NHC and HC proportions for both were considered to be 74.9% and 25.1% [51]. Given this, the excess dietary energy that would be available, were plant sources used instead of all HC and NHC animal-sourced ingredients, for dog, cat and human diets, would be as follows. The E_A_ quantities were provided in Table 4. For human diets, the NHC fraction remains 0.


$$\begin{array}{l} \text{Dog}\;\text{food}\;:\;EA_{dogs}\;\text{x}\;[HC\;+\;(NHC\;\text{x}\;1.352)]\;\text{x}\;3.7\\ =330.4\;PJ\;\text{x}\;[25.1\%\;+\;(74.9\%\;\text{x}\;1.352)]\;\text{x}\;3.7=1,544.8\;PJ. \end{array}$$

$$\begin{array}{l} \text{Cat}\;\text{food}\;:\;EA_{cats}\;\text{x}\;[HC+(NHC\;\text{x}\;1.879)]\;\text{x}\;3.7\\ =39.1\;PJ\;\text{x}\;[25.1\%+(74.9\%\;\text{x}\;1.879)]\;\text{x}\;3.7=239.9\;PJ. \end{array}$$

Dogandcatfood:1,544.8PJ+239.9PJ=1,784.7PJ.

$$\begin{array}{l} \text{Human}\;\text{food}:EA_{humans}\;\text{x}\;[HC+(NHC=0)]\;\text{x}\;3.7\\ =4,940.8\;PJ\;\text{x}\;[100\%+(0)]\;\text{x}\;3.7=18,281.0\;PJ. \end{array}$$

Given this excess dietary energy, and recalling that a million global citizens could be fed per 3.44 PJ of dietary energy (Table 2), the numbers of additional people that could be fed through consuming this energy directly in the form of plant-based ingredients (i.e., within a vegan diet), are provided in Table 20.

**Table 20:** Proportion of the 2018 world human population who could be fed with food energy savings associated with vegan diets.

Vegan diet	Food energy savings (PJ)	People fed (millions)	% of 2018 world population	Regions that could be fed
**Dog food**	1,544.8	449.1	5.8	European Union
**Cat food**	239.9	69.7	0.9	France or the UK
**Dog + cat food**	1,784.7	518.8	6.8	Europe & Central Asia
**Human food**	18,281.0	5,314.2	69.2	Every single nation or collective region on Earth

Note: In all cases the numbers of additional people who could be fed, exceeded the populations within the regions listed as examples. These are based on 2018 populations and World Bank [79] regional definitions.

Hence, compared to using vegan diets to feed people globally, the use of nutritionally-sound vegan dog food would free sufficient food energy to feed 0.08 times as many people. Nutritionally-sound vegan cat food would free sufficient food energy to feed 0.01 times as many, and use of vegan dog and cat food combined would free sufficient food energy to feed 0.10 times as many people–i.e., one tenth as many people who could be fed using the food energy saved, if all people globally transitioned on to vegan diets.

### Additional dogs and cats who could be fed using food energy savings

The excess dietary energy available within meat-based pet food within the US in 2020 and globally in 2018 was provided in Tables 19 and 20. Given that a million dogs (within the US, and globally) can be fed per 2.06 PJ of dietary energy, and that that a million cats (within the US, and globally) can be fed per 0.34 PJ of dietary energy (Tables 1 and 2), the numbers of additional dogs and cats who could be fed with the excess dietary energy available within pet food were calculated (Table 21). For example, the 265.7 excess PJ available within US dog food could feed 129.0 million extra dogs, or 149.5% of the 2020 US dog population. In all cases (dogs, cats, US in 2020, globally in 2018), around 150% - 190% of the existing populations could be fed, with the food energy saved if all dogs and cats within these groups were transitioned on to nutritionally-sound vegan diets.

**Table 21:** Proportion of the 2020 US and 2018 world dog and cat populations who could be fed with food energy savings associated with vegan diets.

	2020 US population			2018 world population		
Vegan diet	Food energy savings (PJ)	Dogs or cats fed (millions)	% of 2020 US population	Food energy savings (PJ)	Dogs or cats fed (millions)	% of 2018 world population
Dog food	265.7	129.0	149.5	1,544.8	749.9	159.2
Cat food	34.3	100.9	165.1	239.9	705.6	189.2

Note: Food energy savings associated with dog or cat food are used to provide additional numbers of dogs or cats who could be fed respectively. i.e., in each case the species remains unchanged.

## Discussion

### Populations of dogs and cats

As noted previously, AVMA [36] estimates of the US dog and cat populations were used, to provide the most conservative estimates of the impacts of dog and cat food. The true numbers of animals–and hence, impacts of pet food–may be substantially higher than estimated in this study. The AVMA estimated the 2020 US dog population at 86.3 million, and the cat population at 61.1 million. The other main population data comes from the *APPA National Pet Owners Survey* [38]. Based on the number of households owning pets, and average numbers of pets per household [38], the US dog population can be estimated at 107.6 million (24.7% higher than the AVMA estimate), and the cat population at 120.1 million (96.6% higher).

For global populations, a wide range of secondary sources exist, but they rarely provide complete global estimations for dogs or cats kept by guardians–as distinct from strays–or utilise reliable primary sources. The 2018 estimations of 471 million dogs, and 373 million cats kept [12], were the most recent global estimations that could be sourced.

However, both within the US and globally, many millions of stray, free-roaming or community-fed animals also exist. Smith *et al*. [80] estimated the worldwide population of domestic dogs at approximately 700 million, with around 75% classified as free-roaming. Belsare and Vanak [81] reported the global dog population as ∼ 0.7–1 billion. Osborn [82] reported that there are an additional 480 million stray, and 100 million wild cats. This current study focused only on animals kept by guardians, whose diets could be studied with greater accuracy. Globally however, millions of additional dogs and cats are fed by people or scavenge for food scraps, with these varied diets also including some livestock produce. Any use of meat-based diets purchased and fed by people, such as those caring for stray dogs or feral cat colonies, further increases livestock production and consumption levels. Hence, the true consumption levels of livestock animals–both in the US, and globally–and the true environmental impacts of dog and cat food, are considerably greater than those conservatively estimated in this study.

#### Dietary energy requirements of dogs, cats and people

For human populations within the US and globally, energy requirements for average men and women aged 19–64 were applied [46]. These were the male and female categories with the greatest, or equal greatest, energy requirements. Actual requirements among people vary based on demographic differences in age, sex, body weight, climate, exercise level, medical conditions and other factors. On average, actual male and female dietary energy requirements will normally be lower than those used in this study, meaning that human dietary energy needs have been over-estimated, compared to those of dogs and cats. This also means that actual consumption of livestock by dogs and cats will be greater than the proportions conservatively estimated within this study, and that the sustainability benefits of nutritionally-sound vegan canine and feline diets, are greater than those calculated.

The FAO data [47] revealed significant differences in the levels of animal produce consumption within human diets (28.7% in the US, versus 18.7% globally). This is consistent with much higher animal produce consumption within high income nations, compared to lower income regions [83]. This is consistent with the lower proportion of NHC ingredient consumption within US pet food compared to more expensive HC ingredients, than was identified globally.

#### Animal by-product use within society

Until recently, accurate information on the level of NHC animal-based ingredients within pet food has been sparse. In 1997, Halpin *et al*. [63] surveyed large petfood manufacturers. They reported that meat by-products comprised around 25–40% of dog foods, and 35–50% of cat foods. Within the current study using 2018–2019 data sourced from 68.3% of US retail pet food sales, NHC sources (primarily, ABPs), provided 52.6% of dog food ingredients, 50.8% of cat food ingredients, and 52.1% of dog and cat food ingredients overall.

It has often been assumed that the use of ABPs within pet food effectively recycles by-products of the human food production system that would otherwise be wasted (e.g., [21, 84])–i.e., that this is environmentally beneficial. One noteworthy finding of this study, is that this assumption has been incorrect.

This study found that NHC sources were less efficient than HC sources, requiring more livestock animals to produce– 1.352 times as many, for dog food, and 1.879 times as many, for cat food. This is consistent with a study by Rushforth and Moreau [85], who found that using lean meat within dog food was better—in terms of environmental impacts—than using offal.

**Fig 4:**
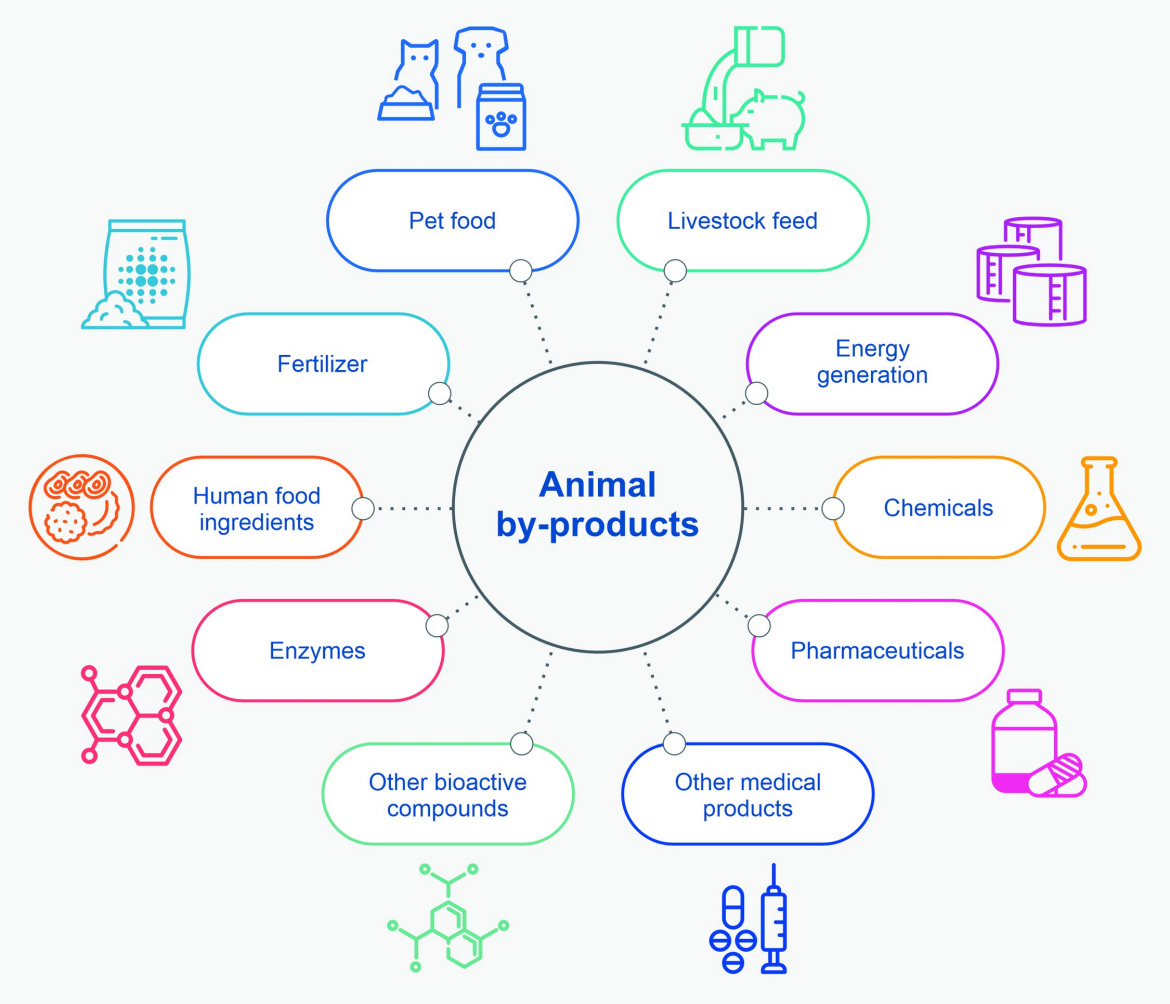
Main social applications of animal by-products. After Toldrá *et al*. [88].

Rather than being wasted, if not consumed within pet food, all meat ingredients, ABPs and their derivatives, would normally be consumed either directly by people, or within other sectors of society [86, 87] (Fig 4).

ABPs account for the majority of slaughtered animal carcases for agricultural species such as cattle (66%), pigs (52%) and lambs (68%) [64]. Around two thirds of these ABPs are directly edible by humans [89]. Organs such as the liver, kidney, heart, brain, intestine, tongue and spleen are HC, and are also termed ‘organ meats’ or ‘variety meats’ [64]. The great majority of animal-sourced material is edible if cleaned, handled and processed appropriately. In developing nations, most of the soft tissues are consumed by people. These include livers, hearts, brains, lungs, the thymus and pancreas, testicles, tongue and gizzard, etc. Classification as HC or NHC often depends on cultural factors such as purchasing power and economics, custom, tradition, food habits, hygiene, availability and religious beliefs of consumers.

ABPs usually considered inedible by humans include hides, skins, ears, snouts, gallbladders, foetuses, hoofs, horns, hair and bristles, etc. Some apparently NHC ingredients are actually used within the human food industry (e.g., edible tallow, blood sausages or pudding, sausage skins, gelatin and defatted meat tissue). Hence, such ingredients are actually HC [63]. Furthermore, many initially inedible ABPs may be converted to edible products through technological innovations. For example, poultry feathers and heads, skin trimmings, fish scales, horns and hooves can all be converted into protein hydrolysates through acidic/alkaline/enzymatic hydrolysis. These protein hydrolysates are used as protein fortifying agents for concentrated soups and beverages, and within solid and liquid seasoning [64].

ABPs may also be classified into principal by-products–directly harvested from the animals, e.g., hides and skins, bones, blood, hoofs and horns, and secondary by-products–derived from these, e.g., bone meal, fat, intestinal linings, etc. ABPs may be further converted via rendering into meat meals and fats. Meat meal is the major secondary by-product produced and is an important components of livestock feeds for pigs and poultry [64]. Bone meal is also important within livestock feeds [90].

ABPs may also be used to create a wide variety of industrial, consumer and medical products. These may include clothing, carpets, blankets, upholstery, rubber, adhesives, lubricants, abrasives, paints, pesticides and fertilizers, soaps, other cosmetics and personal care products, shampoos, detergents, foaming agents and musical strings. They also include a variety of medical products such as pharmaceuticals, surgical sutures, prosthetic materials, collagen sheets, burn dressing, dialyzing membrane, heparin, numerous exogenous hormones, and others [63, 64, 91]. Even bones are used to create a wide variety of products, including tallow, dicalcium phosphate, bone meal, glue and gelatine, and bone morphogenic protein for use within human facial, dental and aesthetic surgeries [64].

Even parts which are contaminated and decomposed may be suitable for products such as fertilizers and soil conditioners. Components such as urine, faeces, ruminal contents, blood, meat and fat trimmings, can be used to create biogas, which may then be burnt to help power abattoirs, power stations or other facilities [64]. Such use of animal parts within energy production may be set to increase further, with ABPs potentially being used within sustainable jet fuel. Forthcoming European legislation could require a majority of aviation fuel to be sustainably sourced. Such developments could lead to scarcity of ABPs for use within pet food [91].

In fact, very little of any animal carcass is wasted. Hence the slaughtering industry colloquialism that, “the packer uses everything but the squeal” [64]. The pet food industry is, in fact, a minority user of animal-based ingredients. Halpin *et al*. [63] estimated that only approximately 25% of all ABPs produced in the US are used within pet foods.

In short, ABPs and their derivatives are used within pet food as protein sources, because they’re considerably cheaper than HC ingredients such as meat. This is not done to ‘recycle’ produce that would otherwise be wasted. Were animal-based ingredients not used within pet foods, they would be consumed in a wide variety of other social sectors. Their consumption within petfood increases overall demand for ABPs–and hence, the number of livestock animals required to provide them.

#### Various environmental impacts

The reductions of various environmental impacts associated with the livestock sector, that could be achieved through transition to nutritionally-sound vegan diets for dogs, cats and people, were shown in Tables 17 and 18. Although the relative numbers of livestock animals required to fulfil the E_A_ needs of humans was much greater than those of dogs and cats (Tables 10 and 11), the diets of dogs and cats have much higher proportions of animal products (Tables 3 and 4), which increases their relative environmental impacts. Accordingly, whilst the greatest reductions in environmental impacts are achievable through transition of humans to vegan diets, the benefits achieved by transitioning dogs in particular, often appear around one quarter to one third of the benefits that could be achieved through human dietary change, at least in the US (Table 17).

At global consumption levels (Table 18), the benefits achieved by vegan pet food reduce, due to lower per capita levels of pet guardianship when compared to the US. This is partly offset due to the higher use of NHC ingredients (74.9%) globally, compared to the US (∼50%). As demonstrated previously, greater NHC use requires more average livestock animals, increasing environmental impacts. Hence, environmental impact reductions achieved by vegan diets for dogs in particular are still significant, compared to reductions achieved by vegan diets for humans. They generally achieve between one fifth and one tenth of the latter effect.

*Consistency with prior studies*. The environmental impacts of dog and cat food demonstrated within this study were very considerable. This concurs with results of other studies within this field. Okin [7] calculated that pet food was responsible for 25–30% of the environmental impacts of the livestock sector within the US, such as the use of land, water, fossil fuels, eutrophifying phosphates, and biocides. The current study estimated the proportion of livestock consumption–and hence livestock-associated environmental impacts–attributable to the diets of US dogs and cats collectively, to be 20.0%. Key differences between these studies are that Okin did not account for the proportion of NHC ingredients within dog and cat diets, and the inefficiencies of producing these ingredients–which requires more livestock–compared to HC ingredients. Additionally, Okin calculated the E_A_ of humans was 20%, but used only data for red meat, poultry and fish, published in 2012. This data excluded animal produce such as eggs and cheese, which are included within diets of humans, dogs and cats [49], and milk, which is included within human diets, and is associated with substantial environmental impacts. After analysing the more complete FAOSTAT supply dataset [47], the current study calculated the E_A_ of US people in 2020, to be 28.7% (Table 3). Furthermore, when apportioning calories between pet and human diets, Okin used consumption data for people, but only energetic needs for dogs and cats. Due to excesses including losses, wastage and overconsumption, actual consumption levels for dogs and cats were therefore underestimated, compared to human levels, lowering estimations of the environmental impacts of pet food. Okin acknowledged this: “An important caveat for the calculations of the relative consumption of pets and humans is that the sources of the data, and mode of calculation, are dramatically different. As a result, their ratios may be systematically biased.” Nevertheless, Okin’s study was an important initial estimation of the environmental impacts associated with dog and cat diets. Okin also concluded that these were very substantial.

Su *et al*. [8] described the concept of the dietary “Ecological Paw Print” (EPP) for dogs and cats. This is equivalent to the human dietary “Ecological Footprint” (EF), and indicates how much productive land is required for an individual or population to maintain itself, and to process resultant waste. These are distinct from total paw- or footprints, which consider requirements for all activities, rather than just diets.

When considering the 27.4 million companion dogs and 58.1 million companion cats in China in 2015, Su *et al*. [8] calculated that the dietary EPP for all dogs and cats was 43.6–151.9 million ha. per year, or 0.82–4.19 ha per year for an average sized dog, and 0.36–0.63 ha per year for a cat. This was equivalent to the dietary EF of 5.1% - 17.8% (70.3–245.0 million) of the Chinese human population in 2015. The annual food consumption of all these dogs and cats was responsible for 2.4–7.5 million tons of carbon emissions, and equivalent to the dietary carbon emissions of 2.5% - 7.8% (34.3–107.1 million) of the Chinese population in 2015.

Similarly, when considering the over 20.3 million companion dogs and cats in Japan, Su and Martens [9] found that the dietary EPP of all dogs and cats was 6.6 million—28.3 million ha per year, comparable to the dietary EF of 4.62 million—19.79 million Japanese people. For an average-sized dog this was 0.33–2.19 ha per year–equivalent to one Japanese person’s dietary EF. The dietary EPP of an average-sized cat was lower, at 0.32–0.56 ha per year. The GHG emissions from Japanese dog and cat food consumption were 2.52 million—10.70 million tons, which was equivalent to the dietary GHG emissions of 1.17–4.95 million Japanese people.

With regard to Dutch companion dogs and cats, the dietary EPP of an average-size dog was 0.90–3.66 ha per year, whereas for a cat, it was between 0.40–0.67 ha per year. The dietary EPP of all Dutch companion dogs and cats was 2.9 million—8.7 million ha per year, equivalent to the entire EF of 0.50 million—1.51 million Dutch people. The GHG emissions from Dutch dog and cat food consumption were 1.09–3.28 million tons, equivalent to the total (i.e., not just dietary) emissions of 94,000–284,000 Dutch people [10].

This demonstrates the capacity for national variation. The dietary EPP of an average companion dog relying on commercial dry food in the Netherlands or in China was considerably greater than in Japan, although for companion cats these were similar among all three nations. Even in Japan, however, the dietary EPP of an average companion dog was equivalent to the dietary EF of an average Japanese person. And in all cases, dietary EPPs of companion dogs and cats equalled significant proportions of total human dietary EFs.

Vale and Vale [30] calculated dietary EPPs for small, medium and large dogs of 0.18, 0.27 and 0.36 ha/year, and for cats, of 0.3 ha/year. These were usually slightly lower than calculated by Su *et al*. [8], Su and Martens [9], and Martens *et al*. [10]. However Vale and Vale excluded footprints produced by ingredient processing, diet manufacturing, packaging and transporting. Using data from North Western Europe, Leenstra and Vellinga [92] estimated a dog paw print of 0.2 ha, and a cat paw print of 0.1 ha. However, the relatively high crop yields within this region may have lowered paw prints, compared to some other world regions.

The Brazilian canine population of 52.2 million is one of the world’s largest. Pedrinelli *et al*. [11] studied the diets of 618 Brazilian dogs and 320 Brazilian cats. An average canine diet was responsible for 828.37 kg of CO_2_eq annually (dry diets) or 6,541 kg of CO_2_eq (wet diets), equivalent to 12.4 or 97.8% respectively of the emissions of a Brazilian person (6.69 t CO_2_eq annually). For the entire Brazilian canine population, dog food-associated emissions were 0.04–0.34 Gt CO_2_eq annually, or 2.9–24.6% of Brazil’s total estimated emissions (1.38 Gt annually). This study demonstrated the markedly greater impacts of wet diets compared to dry diets.

Alexander *et al*. [93] estimated the environmental impacts associated with global dry pet food production. This was estimated to create 56–151 Mt CO_2_ equivalent emissions (1.1% − 2.9% of global agricultural emissions), and to use 41–58 Mha of agricultural land (0.8–1.2% of global agricultural land), and 5–11 km^3^ of freshwater (0.2–0.4% of agricultural water extraction). However, they noted that this was based solely on dry food data, which constituted only 79% of US pet sales. Furthermore, they used an economic valuation to consider the impacts of ABPs, thereby substantially underestimating the environmental impacts of ABPs, which have low economic value. As demonstrated in this current study, ABPs require more, not less, average livestock animals, and have greater environmental impacts, than the use of HC ingredients. Alexander and colleagues also did not account for pricing variations globally, but similar pet food may be priced very differently, in different world regions. Finally, they assumed that global pet food volumes were weighted equally according to US dog (78%) and cat (22%) energy consumption [7], although dog and cat populations, and their relative proportions, vary substantially between countries. Hence, their results were impacted by substantial underestimations and uncertainties. Even so, they also estimated very significant environmental impacts, associated with global dry pet food production.

*Additional impacts and future trends*. It must be acknowledged that although the environmental impacts of dog and cat food revealed by all of these studies and this current study are considerable, they do not capture all impacts. Impacts are associated not only with primary production of animal- and plant-based ingredients, but with their processing, shipping, retail, storage, cooking, dishwashing and waste disposal. Many of these stages also include transportation impacts [59].

Impacts of pet food are also likely to increase in future, due to the rapid increases in the global companion dog and cat populations over decades [93], driven partly by human population growth, and facilitated by the economic development of some nations, which increases disposable incomes, and capacity to support pet guardianship. This is demonstrated by pet food sales trends. From 2022 to 2027, the global market for pet food ingredients is expected to increase from $32.2 - $44.5 billion–a compound annual growth rate of 6.7% [51].

### Study limitations and future research suggestions

To determine the impacts on sustainability indicators of animal produce consumption, and the benefits achievable through transition to vegan diets, for dogs, cats and people, a number of assumptions were required at various stages. In some cases, provision of additional data could refine the accuracy of subsequent calculations.

#### Dietary energy requirements of dogs, cats and people

The first assumption related to dietary energy requirements of dogs, cats and humans. The energy needs for dogs [43] and cats [45] were calculated using body weight averages published by Bermingham *et al*. However, dog breeds vary dramatically in size [42], resulting in markedly different MER requirements ([43], Table 3). Energy requirements also vary significantly with husbandry type and activity level, with requirements greatest in racing dogs, followed by working and hunting dogs, and finally, by pet and kennelled dogs. Very young or old dogs, or those who are pregnant, lactating or unwell, may also have significantly different energy requirements [94]. Although MERs appear equal between sexes, they are lower in neutered compared with sexually intact dogs [43]. For the purposes of this study, the average MER of dogs calculated by Bermingham *et al*.–partly on the basis of BW, was extrapolated to all US dogs. However, this is only an approximation of the true MER of all US dogs. As Bermingham *et al*. noted, “estimating maintenance energy requirements based on BW alone may not be accurate … predictions that factor in husbandry, neuter status and, possibly, activity level might be superior.” They also noted more information is needed about the nutrient requirements of older dogs, and of giant and toy breeds.

Similarly, the average energy requirements for domestic cats, calculated by Bermingham *et al*. [45], were extrapolated to all US cats. This was also an approximation. As stated by Bermingham *et al*., “maintenance energy requirements were significantly affected by weight, sex and neuter status, age and methodology”.

One key consideration is that the average body weight of dogs and cats is increasing over time, due to the increasing prevalence of overweight and obesity in kept dogs and cats. Hence the average body weights of dogs and cats can be expected to have increased significantly since the canine and feline averages were published by Bermingham *et al*. in 2014 [43] and 2010 [45] respectively. In both species weight gain results in significant increases in daily energy requirements [43, 45]. Hence the energy requirements estimated by Bermingham *et al*. for US dogs and cats, used within this study, probably significantly underestimated the true energy requirements of dogs and cats today. Updated demographic data for humans, dogs and cats, would allow more precise characterisations of these populations, and more accurate calculations of their dietary energy needs.

#### Total energy from animal sources (E_A_), consumed by dogs, cats and people

When calculating the proportion of human dietary energy attributable to animal produce (E_A_), FAOSTAT data [47] were used to provide separate estimates for the US, and globally. The daily calories provided did not include quantities exported, fed to livestock, used for seed, put to manufacture for non-food uses, or losses during storage and transportation. Nevertheless, they remained substantially higher than daily energetic needs, both within the US, and globally. The excess calories were assumed to have been lost at later stages, e.g., retail, wasted or overconsumed. Comparative data on such excess levels within the diets of dogs, cats and people were not available; hence, these were assumed to occur at equal proportions within all of these dietary groups, allowing them to be discounted when considering the proportional consumption of average livestock animals, among dogs, cats and people. In reality however, such excess proportions may not be equal. Data on actual levels and differences between dietary groups, would allow refinements of the proportional livestock consumption estimates provided by this study.

When calculating the proportion of dog and cat dietary energy attributable to animal produce (E_A_), data from Okin [7] were used. However, these E_A dogs_ and E_A cats_ proportions were calculated by Okin using the E_A_ within US premium and non-premium (‘market leading’) dog and cat foods, weighted by the proportions of US consumers choosing each. E_A_ was significantly higher within the premium brands. These data allowed accurate prediction of livestock consumption within US pet foods, but would have been less accurate when considering pet food globally. Due to lower average wealth, a higher proportion of consumers globally would have been likely to choose cheaper, non-premium brands, with lower E_A_s. This factor could decrease the relative impacts of pet food globally, compared to those predicted by US figures. To provide more accurate estimations of global pet food E_A_ fractions, global data could be sought and utilised if available, concerning the E_A_ fractions within premium and non-premium brands, and the proportion of consumers choosing to purchase each.

#### Animal-based ingredients used to feed people, dogs and cats

When considering the various animal- and non-animal sourced ingredients within dog and cat food, the consumption data analysed for US pets were unusually detailed, but were not perfectly so. The DIS data [49] studied directly represented 68.3% of US retail pet food sales from July 2018 –June 2019. These were extrapolated (multiplied by 1/0.683) to estimate all of US retail pet food sales. Hence, the data used covered just over two thirds of the market. It also included major pet food companies. Accordingly, this extrapolation was probably quite accurate, although data covering the entirety (without extrapolation) of US retail pet food sales would have been even more accurate. Unfortunately, such data were not available within the US, nor globally, meaning that these US results also had to be extrapolated to pet food globally.

#### Consumption of HC and NHC ingredients within dog and cat food

For each HC and NHC group, it was necessary to determine the proportion of average livestock animals (carcasses), that produced HC or NHC components. This allowed comparison of the efficiency of average livestock animals, at providing these two ingredient groups. To achieve this, the largest subgroup within each group was used as a proxy for the entire group. As noted, just under half of dog and cat food was provided by HC components, and just over half, by NHC components. Meat was used as a proxy for the HC group (comprising 66.2% of this group, for dog food, and 50.0%, for cat food), and animal meal as a proxy for the NHC group (comprising 88.1% of this group, for dog food, and 74.0%, for cat food). Given the proportionate sizes of these subgroups, extrapolation to cover each entire group seemed reasonable. However, accuracy could be increased by considering the full range of ingredients used. Livestock (carcass) proportions for all species supplying each of those ingredients could be sought where available, and could be included within averages weighted by consumption. This would allow more accurate determination of the proportion of average livestock animals, that produced HC or NHC components.

#### Attribution of energy consumption to HC and NHC components

Having calculated the relative efficiencies of average livestock animals at providing HC and NHC dietary components, the E_A_ dietary fraction was then appropriately apportioned to these HC and NHC components, for dog and cat food. However, this required assuming that the E_A_ dietary energy was evenly distributed across the animal-sourced ingredients used.

In reality, the energy density of different animal-sourced ingredients is not uniform. However, neither do they seem widely distributed. As noted within the Methodology, the energy density of a variety of meats including poultry and fish, are around 200 kcal/100 g [50]. Hence, this assumption does appear reasonable. It was also hoped that any differences would average out to some degree, across the ingredients used. For dog food, 52 animal-sourced ingredients existed, represented by nine ingredients within the meat proxy group, and 14 ingredients within the animal meal proxy group. For cat food, 47 animal-sourced ingredients existed, represented by seven ingredients within the meat proxy group, and 11 ingredients within the animal meal proxy group.

Whilst this study has provided a reasonable estimation based on averaging, future research accounting for differences between these ingredients is recommended to provide more accurate estimates. Actual energy densities could be sought and used where available, within weighted averages. Energy densities of some ingredients (especially HC) are available via sources such as the USDA Food Data Central database [95].

#### Environmental sustainability indicators

Calculation of environmental impacts of plant- versus animal-based ingredients relied on 2009–2011 averages for 52 plant- and animal-sourced food ingredients, using globally-sourced data [55]. There are very few such comprehensive data sets, and this is one of the most recent. These calculations could be updated in future as more recent data sets become available.

From these data, production volumes for food purposes for all ingredients were used to calculate weighted averages. However, whilst this is accurate for society as a whole, within the different dog, cat and human dietary groups, consumption proportions of the various ingredients would vary. Hence, the environmental impact estimates derived could be refined through consideration of actual ingredient consumption proportions, within these different dietary groups.

Finally, the attribution to dog and cat food of specific proportions of global livestock animal consumption–and hence, of global environmental impacts associated with the farming of those animals, relied on analysis of ingredients within the diets of US dogs, cats and humans. In reality, there will be regional and national differences in ingredient consumption, across all dietary groups, and global extrapolation will not be entirely accurate.

Despite such international variations, several factors made it reasonable to use US data as the basis for global extrapolation. Firstly, with over 86 million owned dogs and 61 million owned cats, the US was the country with the largest national populations of these animals. It comprised around 18.3% of the world’s 471 million owned dogs, and 16.4% of the world’s 373 million owned cats (based on US 2020 figures, and global 2018 figures). With respect to ingredients consumed, the US was the only region with very detailed data concerning dog and cat food ingredient consumption levels, predicting national consumption.

Finally, for US dog and cat food, NHC components comprised 52.6% and 50.8% of all animal-sourced ingredients respectively. In comparison, the global consumption of meat meal, ABP meal and animal digest within pet food (comprising all NHC ingredients) in 2020, comprised 17,113.1 kT, or 74.9% of the 22,841.1 kT total meat and meat products consumed within pet food (T = US ton) [51]. Hence, such NHC ingredients comprised a significantly higher proportion of pet food globally, than within the US. This probably occurred because such ingredients are cheaper, and the US is wealthier than most other countries. The global pet food ingredients market was worth $32.2 billion in 2022, with the North American market worth 36.2% of that–the largest regional share ([51], Table 2)–despite including only ∼16–18% of the world’s owned dogs and cats, as noted. Hence, US pet food has a significantly higher HC component, than pet food globally. But as calculated previously, HC ingredient provision is more efficient than NHC provision. It requires fewer average livestock animals to produce, decreasing environmental impacts. Hence, per kg of dog and cat food, environmental impacts would have been significantly lower in the US, than the global average. As noted previously, approximately 8.65 million tons of animal- and plant-based ingredients were used within US dog and cat food annually, from mid 2018–2019 [96]. Globally, 53.49 million tons of ingredients were used in pet food, in 2019 [51]. Hence, around 83.8% of consumption globally, was from regions where environmental impacts were significantly higher, than estimated for the US in this study. Accordingly, despite the various assumptions made–frequently based on the use of averages, the estimates of environmental impacts for dog and cat food, derived in part through extrapolation from US data, are very conservative. The true global environmental impacts of dog and cat food, are probably significantly higher than estimated in this study. More accurate estimations of impact in non-US regions and globally, could be derived through consideration of actual levels of NHC ingredient use within pet food, where these are available.

#### Additional people who could be fed using food energy savings

It was noted that for every 1.0 J of animal-sourced HC ingredients consumed, an average of 3.7 J of excess dietary energy was lost during conversion from plant- to animal-sourced ingredients. This 3.7 J was used to calculate additional food energy that would become available, were dogs, cats or people transitioned on to vegan diets. However, this 3.7 J was calculated by considering the average loss-adjusted feed conversion ratio for beef+lamb, pork, and poultry, weighted by their relative availability in the diets of American people [60]. Although these meat products comprise the great majority of meat consumed by people, as well as by dogs and cats, the average diets of American people, dogs and cats all include additional animal-sourced ingredients, and the proportions of these animal-sourced ingredients are not uniform. Accordingly, whilst 3.7 J covers most of the meat consumed, it remains only an approximation for the excess energy inherent within the animal-sourced ingredients within these diets. More accurate estimations could be derived by considering a wider range of animal-based ingredients, and their different consumption levels, within dog, cat and human diets.

### Recommendations for reducing environmental impacts

Pet diets are not the only aspects of pet guardianship with environmental consequences. As noted by Su *et al*. [8], companion animals also need water, living spaces, entertainment, health care and other resources and services, which substantially increase their environmental impacts [97]. Nitrogenous waste products from excreta also increase environmental impacts [98]. Yavor *et al*. [99], for example, found that the urine and faeces of an average dog has a climate change and freshwater eutrophication potential of around 8,200 kg CO_2_eq and 5.0 kg Peq, respectively. However, the effects of diets exceed those of most other sectors. With respect to GHGs, for example, the food sector and livestock sectors are respectively responsible for 35% and 20% of all GHGs globally [3]. As shown in this study, the effects of meat-based dog and cat food, are marked. Others (e.g., [7, 100]) have suggested that animals with lower dietary requirements (e.g., cats, small dogs), or herbivorous animals (e.g., horses, rabbits and rodents), could be kept instead. This has some merit. A systematic review of 29 studies by Birmingham *et al*. ([43], Table 3) found that the average MERs of dogs varied depending on breed size, from 206 (toy) to 3,020 (giant) kcal/day. As noted, the overall canine average of 1,351 kcal/day has been used in this study. Similarly, Su and Martens [9] found that a large dog’s dietary EPP was equivalent to that of around nine small dogs, or 12 cats.

Improvements could also be sought to improve efficiency and minimise wastage within pet food manufacturing processes, packaging materials and transportation methods [32]. Dietary formulation is important–Pedrinelli *et al*. [11] demonstrated that wet food diets had far greater environmental impacts than dry diets. It is also important to minimise overconsumption [84] and wastage of food. Due to excessive consumption, over 50% of pet dogs in various geographical areas are now obese [101]. Some studies have demonstrated similar results for cats [102]. Overfeeding and food wastage further increases livestock consumption and associated environmental impacts.

However, as shown in this study, nutritionally-sound vegan dog and cat diets clearly offer major environmental sustainability benefits. These are usually formulated using terrestrial plants, but yeast/fungi or seaweed-based diets may also be available now or in the future. At least one pet food company, for example, combines yeast- and plant-based ingredients, supplemented with all essential canine nutrients, to produce canine ‘performance’ and ‘maintenance’ kibble formulations, with Metabolizable Energies ranging from 3,435–3,678 kcal/kg [103]. These compare favourably to those found within other dog foods [104]. Many other nutritionally-sound vegan dog and cat foods already exist [100], and this sector is growing rapidly [51]. It is likely that the most effective way to reduce environmental impacts associated with guardianship of companion animals, is to transition them on to nutritionally-sound vegan diets.

## Conclusions

The adverse environmental impacts of the livestock sector have been well-studied (e.g., [1–3]), and accompanied by many calls for transitioning to plant-based diets (e.g., [5, 6]). The impacts on climate change alone, justify such action. The livestock sector contributes 20% of all anthropogenic GHGs [3], and in 2023 the Intergovernmental Panel on Climate Change [105] noted that “Climate change is a threat to human well-being and planetary health (*very high confidence*). There is a rapidly closing window of opportunity to secure a liveable and sustainable future for all (*very high confidence*).” In response, United Nations Secretary-General Antonio Guterres stated, “Our world needs climate action on all fronts—everything, everywhere, all at once” [106]. To date, corresponding calls for a transition to plant-based diets have largely focused on people. However, dogs and cats are also major consumers of livestock animals. The global population of kept dogs and cats is around 10% of the human population, and the numbers of stray or free-roaming animals are even higher.

Until recently, assumptions that dogs and cats could not thrive on vegan diets probably prevented serious calls for similar dietary change among these groups. However, a sizeable and rapidly-growing body of evidence has now shown that both dogs and cats can thrive on nutritionally-sound vegan diets. Such results are evident within both canine (nine studies: [16–24]) and feline (four studies: [23, 25–27]) studies of health outcomes. Furthermore, their behavioural needs and welfare are not compromised by such diets [28]. Accordingly, it is now important to compare the environmental impacts of conventional meat-based diets, among dogs, cats and humans, and to compare the benefits that would be expected to accrue, were each group transitioned on to nutritionally-sound vegan diets.

This study demonstrated that the benefits of such a transition would be substantial, for all of these populations. The most accurate, recent dog and cat population estimations dated from 2020, for the US, and from 2018, for global populations. The US populations in 2020 were estimated to include at least 86 million dogs, 61 million cats, and 329 million people. The global populations in 2018 were estimated to include at least 471 million dogs, 373 million cats, and 7.68 billion people. The relative consumptions of average livestock animals by these groups were estimated within the US as: dogs– 17.7%, cats– 2.3%, humans– 80.0%, and globally as: dogs– 7.7%, cats– 1.2%, humans– 91.1%. These differences reflected significantly greater pet guardianship in the US, compared to the global average, consistent with the US being a wealthy, highly developed nation, with relatively high disposable incomes available to support pet guardianship.

If all of these groups transitioned to nutritionally-sound vegan diets, the numbers of terrestrial livestock animals spared from slaughter annually were estimated to be (in billions), in the US: dogs– 1.7, cats– 0.2, humans– 7.8, and globally: dogs– 6.0, cats– 0.9, humans– 71.3. The numbers of aquatic animals killed for food annually are far higher, and the use of nutritionally-sound vegan diets would also save billions of aquatic animals, in all dietary groups.

Considering environmental impacts on land and water use, emissions of GHGs, acidifying and eutrophifying gases, and the use of biocides, very substantial impact reductions were associated with the use of nutritionally-sound vegan diets, in all dietary groups. With respect to land use, for example, if implemented globally such diets would free up land larger than the areas of the following nations: dogs–Saudi Arabia or Mexico, cats–Japan or Germany, humans–Russia–the world’s largest country, combined with India. With respect to water use, such diets would save freshwater volumes greater than all renewable freshwater in the following nations: dogs–Denmark, cats–Jordan, humans–Cuba. With respect to GHGs, such diets would reduce GHGs by amounts greater than all GHG emissions from following nations: dogs–South Africa or the UK, cats–Israel or New Zealand, humans–India or the entire EU.

The numbers of additional people who could be fed using food energy savings associated with the global implementation of nutritionally-sound vegan diets among kept dogs, cats and people exceeded the 2018 human populations of the following nations: dogs–the entire European Union, cats–France or the UK, humans–every single nation or collective region on Earth, as defined by the World Bank [79]. When considering the numbers of additional dogs and cats that might alternatively be fed using these food energy savings, in all cases (dogs, cats, US in 2020, globally in 2018), around 150% - 190% of the existing populations could be fed. All of these estimates were conservative. Multiple factors mean the true benefits achieved by transitioning dogs and cats on to nutritionally-sound vegan diets, are likely to be significantly higher.

By far the largest benefits were associated with vegan diets for people. However, in the US, the benefits achieved by transitioning dogs in particular, often appeared around one quarter to one third of the benefits achievable, through human dietary change. Globally, vegan diets for dogs generally achieved between one fifth and one tenth of the latter effect. The relatively greater impacts of dog and cat diets within the US, were most likely due higher levels of pet guardianship than global averages. They indicate the likely future benefits of vegan diets for dogs and cats in other nations, as these similarly develop, making similar levels of pet guardianship financially possible. Per capita pet guardianship is steadily increasing in most nations–including the US. Hence the relative environmental impacts of conventional meat-based pet diets are likely to be even higher in the future, than indicated by the 2018 (global) and 2020 (US) timeframes of this study.

Hence, it is clear that substantial proportions of the impacts of the livestock sector globally, are due to conventional meat-based dog and cat food. The impacts of pet food should not be discounted, when considering environmental impacts of diets. Conversely, great benefits for environmental sustainability can be realised through the use of nutritionally-sound vegan diets for dogs and cats, as well as for people.

## Supporting information

S1 File (ZIP)
