# Supplementary material for: The relative benefits for environmental sustainability of vegan diets for dogs, cats and people
Source: PLoS One. 2023 Oct 4;18(10):e0291791. doi: 10.1371/journal.pone.0291791 (PMC10550159; doi:10.1371/journal.pone.0291791)
Supplement: S1 File — (ZIP) [file pone.0291791.s001.zip › S1 Table.docx]

**S1 Table. Environmental sustainability indicators assessed.** After Poore and Nemecek [55].

| **Indicator** | **Emissions/Uses** |
| --- | --- |
| Land Use x Occupation Time | Seed, on- and off-farm arable and permanent crops, fallow land, temporary pasture, permanent pasture |
| Freshwater Withdrawals | Irrigation, drinking, pond and processing water |
| Scarcity-Weighted Freshwater Withdrawals | Irrigation, drinking, pond and processing water |
| Greenhouse Gas Emissions | CO_2_, CH_4_, N_2_O to air |
| Acidification | SO_2_, NH_3_, NO*_x_* to air |
| Eutrophication | NH_3_, NO*_x_* to air, NO^3–^, NH_4_^+^, P, N to water |
