# Supplementary material for: The relative benefits for environmental sustainability of vegan diets for dogs, cats and people
Source: PLoS One. 2023 Oct 4;18(10):e0291791. doi: 10.1371/journal.pone.0291791 (PMC10550159; doi:10.1371/journal.pone.0291791)
Supplement: S1 File — (ZIP) [file pone.0291791.s001.zip › S10 - S17 Tables - cat food.docx]

**S10 – S17 Tables. Animal-based ingredients included within cat food.**

Note: Quantities are rounded to the nearest ton. Totals are calculated using exact rather than rounded data.

**S10 Table. Animal meals included within cat food, in tons.** Data source: [49].

| Chicken By-product Meal | 161,028 |
| --- | --- |
| Poultry By-product Meal | 103,207 |
| Meat and Bone Meal | 32,686 |
| Chicken Meal | 21,421 |
| Fish Meal | 17,852 |
| Turkey By-product Meal | 15,695 |
| Salmon Meal | 9,816 |
| Tuna Meal | 2,065 |
| Turkey Meal | 842 |
| Beef Meal | 228 |
| Bone Meal | 162 |
| **Total** | **365,001** |

**S11 Table. Meat included within cat food, in tons.** Data source: [49].

| Chicken | 113,731 |
| --- | --- |
| Organ Meat | 81,861 |
| Turkey | 28,379 |
| Beef | 10,243 |
| Poultry | 4,237 |
| Duck | 220 |
| Lamb | 170 |
| Bacon | 54 |
| **Total** | **238,895** |

**S12 Table. Fats and oils included within cat food, in tons.** Data source: [49].

| Animal Fat | 24,992 |
| --- | --- |
| Beef Fat | 23,851 |
| Chicken Fat | 6,301 |
| Fish Oil | 1,406 |
| Poultry Fat | 1,055 |
| Pork Fat | 160 |
| Salmon Oil | 21 |
| **Total** | **57,786** |

**S13 Table. Animal by-products included within cat food, in tons.** Data source: [49].

| Meat By-products | 87,389 |
| --- | --- |
| Poultry By-products | 25,622 |
| Chicken By-products | 306 |
| Other Animal By-products | 264 |
| Pork By-products | 164 |
| **Total** | **113,744** |

**S14 Table. Animal broths included within cat food, in tons.** Data source: [49].

| Poultry Broth | 34,577 |
| --- | --- |
| Fish Broth | 26,547 |
| Chicken Broth | 16,914 |
| Beef Broth | 1,918 |
| Turkey Broth | 854 |
| **Total** | **80,811** |

**S15 Table. Fishery ingredients included within cat food, in tons.** Data source: [49].

| Fish | 37,792 |
| --- | --- |
| Whitefish | 17,943 |
| Salmon | 17,135 |
| Tuna | 12,411 |
| Ocean Fish | 5,293 |
| Shrimp | 2,710 |
| **Total** | **93,285** |

**S16 Table. Dairy and egg ingredients included within cat food, in tons.** Data source: [49].

| Egg Product | 5,273 |
| --- | --- |
| Cheese | 1,300 |
| Egg | 67 |
| **Total** | **6,639** |

**S17 Table. Other ingredients included within cat food, in tons.** Data source: [49].

| Digest Flavor | 14,397 |
| --- | --- |
| Animal Plasma | 2 |
| **Total** | **14,399** |
