# Supplementary material for: The relative benefits for environmental sustainability of vegan diets for dogs, cats and people
Source: PLoS One. 2023 Oct 4;18(10):e0291791. doi: 10.1371/journal.pone.0291791 (PMC10550159; doi:10.1371/journal.pone.0291791)
Supplement: S1 File — (ZIP) [file pone.0291791.s001.zip › S18 Table.docx]

**S18 Table. Ingredients considered when calculating environmental impacts of dog and cat diets.** Source: Poore and Nemecek [55].

|  | **Animal-based** | **Vegan** |
| --- | --- | --- |
| **Included** | bovine meat (beef herd), bovine meat (dairy herd), lamb & mutton, pig meat, poultry meat, cheese, eggs, fish (farmed), crustaceans (farmed), fish & crustaceans (capture), animal fats, buffalo | wheat & rye (bread), maize (meal), barley (beer), oatmeal, rice, potatoes, cassava, other pulses, peas, nuts, groundnuts, soymilk, tofu, soybean oil, palm oil, sunflower oil, rapeseed oil, olive oil, tomatoes, onions & leeks, root vegetables, brassicas, other vegetables, citrus fruit, bananas, apples, berries & grapes, other fruit, cereals & oilcrops misc. |
| **Excluded** | milk, butter, cream & ghee | wine, coffee, dark chocolate, oils misc., stimulants & spices misc., aquatic plants, cane sugar, beet sugar |
