# Supplementary material for: The relative benefits for environmental sustainability of vegan diets for dogs, cats and people
Source: PLoS One. 2023 Oct 4;18(10):e0291791. doi: 10.1371/journal.pone.0291791 (PMC10550159; doi:10.1371/journal.pone.0291791)
Supplement: S1 File — (ZIP) [file pone.0291791.s001.zip › S2 - S9 Tables - dog food.docx]

**S2 – S9 Tables. Animal-based ingredients included within dog food.**

Note: Quantities are rounded to the nearest ton. Totals are calculated using exact rather than rounded data.

**S2 Table. Animal meals included within dog food, in tons.** Data source: [49].

| Meat and Bone Meal | 500,567 |
| --- | --- |
| Chicken By-product Meal | 201,800 |
| Chicken Meal | 175,953 |
| Beef and Bone Meal | 104,111 |
| Poultry By-product Meal | 91,802 |
| Lamb Meal | 33,893 |
| Beef Meal | 27,465 |
| Fish Meal | 19,071 |
| Turkey Meal | 17,203 |
| Meat Meal | 8,861 |
| Salmon Meal | 8,614 |
| Pork Meal | 4,031 |
| Turkey By-product Meal | 71 |
| Bone Meal | 47 |
| **Total** | **1,193,490** |

**S3 Table. Meat included within dog food, in tons.** Data source: [49].

| Chicken | 470,367 |
| --- | --- |
| Beef | 136,419 |
| Organ Meat | 64,825 |
| Lamb | 58,832 |
| Turkey | 33,941 |
| Poultry | 27,032 |
| Pork | 9,345 |
| Duck | 4,955 |
| Venison | 2,250 |
| Bacon | 1,506 |
| **Total** | **809,473** |

**S4 Table. Fats and oils included within dog food, in tons.** Data source: [49].

| Beef Fat | 123,605 |
| --- | --- |
| Animal Fat | 79,518 |
| Chicken Fat | 42,268 |
| Poultry Fat | 13,459 |
| Fish Oil | 3,417 |
| Pork Fat | 831 |
| Salmon Oil | 772 |
| Bacon Fat | 448 |
| **Total** | **264,317** |

**S5 Table. Animal by-products included within dog food, in tons.** Data source: [49].

| Meat By-products | 47,927 |
| --- | --- |
| Beef By-products | 29,320 |
| Chicken By-products | 26,576 |
| Other Animal By-products | 24,054 |
| Pork By-products | 5,749 |
| **Total** | **133,625** |

**S6 Table. Animal broths included within dog food, in tons.** Data source: [49].

| Chicken Broth | 53,666 |
| --- | --- |
| Beef Broth | 13,108 |
| Poultry Broth | 5,003 |
| Turkey Broth | 3,007 |
| Fish Broth | 499 |
| **Total** | **75,283** |

**S7 Table. Fishery ingredients included within dog food, in tons.** Data source: [49].

| Salmon | 35,568 |
| --- | --- |
| Whitefish | 2,405 |
| Fish | 704 |
| Tuna | 286 |
| Ocean Fish | 2 |
| **Total** | **38,966** |

**S8 Table. Dairy and egg ingredients included within dog food, in tons.** Data source: [49].

| Egg | 23,010 |
| --- | --- |
| Egg Product | 10,322 |
| Cheese | 1,499 |
| **Total** | **34,831** |

**S9 Table. Other ingredients included within dog food, in tons.** Data source: [49].

| Digest Flavor | 27,165 |
| --- | --- |
| Animal Plasma | 697 |
| **Total** | **27,861** |
