# Supplementary material for: The relative benefits for environmental sustainability of vegan diets for dogs, cats and people
Source: PLoS One. 2023 Oct 4;18(10):e0291791. doi: 10.1371/journal.pone.0291791 (PMC10550159; doi:10.1371/journal.pone.0291791)
Supplement: S1 File — (ZIP) [file pone.0291791.s001.zip › S20 Table.docx]

**S20 Table. Relative environmental impacts of animal-based versus vegan food ingredients used in dog, cat and human diets, with percentage reductions achieved by vegan diets**. Data sources: Reijnders and Soret [59] (biocides), Poore and Nemecek [55] (others - 2009-2011 avg).

| **Diet** | **Ingredients** |  | **Land Use (m^2^)** | **Freshwater (L)** | **Str-Wt WU (L eq)** | **GHG (kg CO_2_eq, IPCC 2013)** | **Acid.(kg SO_2_eq)** | **Eutr. (kg PO_4_^3-^eq)** | **Biocides** |
| --- | --- | --- | --- | --- | --- | --- | --- | --- | --- |
| Dog and cat food | Animal-based |  | 32.070 | 929.909 | 30299.781 | 12.350 | 0.084 | 0.063 |  |
|  | Vegan |  | 1.696 | 382.498 | 12970.763 | 1.249 | 0.009 | 0.006 |  |
|  | **Relative impact (W): animal/vegan** |  | 18.911 | 2.431 | 2.336 | 9.887 | 9.646 | 9.700 | 6.000 |
| Human food | Animal-based |  | 20.554 | 739.977 | 23912.038 | 7.771 | 0.052 | 0.037 |  |
|  | Vegan |  | 1.764 | 379.675 | 12742.397 | 1.388 | 0.009 | 0.007 |  |
|  | **Relative impact (W): animal/vegan** |  | 11.649 | 1.949 | 1.877 | 5.599 | 5.746 | 5.396 | 6.000 |
| All | **Relative impact: dog or cat (W)/human (W)** |  | 1.623 | 1.247 | 1.245 | 1.766 | 1.679 | 1.798 | 1.000 |
| All | **Reduction of impact with vegan diet, % reduction** | **Dogs** | 6.090, 85.9% | 0.487, 32.7% | 0.454, 31.2% | 3.022, 75.1% | 2.940, 74.6% | 2.958, 74.7% | 1.700, 63.0% |
|  |  | **Cats** | 5.534, 84.7% | 0.442, 30.7% | 0.413, 29.2% | 2.746, 73.3% | 2.672, 72.8% | 2.688, 72.9% | 1.545, 60.7% |
|  |  | **Dogs + cats** | 6.036, 85.8% | 0.482, 32.5% | 0.450, 31.0% | 2.995, 75.0% | 2.914, 74.4% | 2.932, 74.6% | 1.685, 62.8% |
|  |  | **Humans (US)** | 3.056, 75.3% | 0.272, 21.4% | 0.252, 20.1% | 1.320, 56.9% | 1.362, 57.7% | 1.262, 55.8% | 1.435, 58.9% |
|  |  | **Humans (global)** | 1.991, 66.6% | 0.177, 15.1% | 0.164, 14.1% | 0.860, 46.2% | 0.887, 47.0% | 0.822, 45.1% | 0.935, 48.3% |
